# The saline groundwater legacy of a large buried coastal paleo-estuary

**DOI:** 10.1038/s41467-026-76005-5

**Published:** 2026-07-27

**Authors:** Burke J. Minsley, Holly A. Michael, Maxwell A. Lindaman, Lyndsay B. Ball, Stephanie R. James, JR Rigby, Wade H. Kress, Frank T.-C. Tsai, Bennett E. Hoogenboom

**Affiliations:** 1 Geology, Geophysics and Geochemistry Science Center, U.S. Geological Survey, Denver, CO USA; 2 Department of Earth Sciences, Department of Civil, Construction and Environmental Engineering, University of Delaware, Newark, DE USA; 3 Lower Mississippi-Gulf Water Science Center, U.S. Geological Survey, Baton Rouge, LA USA; 4 Lower Mississippi-Gulf Water Science Center, U.S. Geological Survey, Oxford, MS USA; 5 Lower Mississippi-Gulf Water Science Center, U.S. Geological Survey, Nashville, TN USA; 6 Department of Civil and Environmental Engineering, Louisiana State University, Baton Rouge, LA USA; 7 Present Address: California Water Science Center, U.S. Geological Survey, Sacramento, CA USA

**Keywords:** Hydrology, Geophysics, Water resources, Hydrogeology

## Abstract

Elevated groundwater salinity in coastal regions threatens the beneficial use of fresh groundwater. Coastal groundwater management typically focuses on preventing intrusion from modern sources of seawater; however, past geological processes can also leave a legacy of saline groundwater now hidden in the subsurface. Here, multiple extensive airborne electromagnetic surveys provide detailed evidence of residual salinity from a paleo-estuary filling a late Pleistocene incised valley impacting more than 10,000 km^2^ that is now hidden beneath coastal Louisiana’s deltaic plain. Our results show that the three-dimensional pattern of saline groundwater beneath Louisiana mimics that of near-surface aquifers surrounding the modern Delaware Bay estuary, fingerprinting the signature of the past drowning of a large, incised valley of the Mississippi River following post-glacial sea-level rise. These findings demonstrate a new framework for understanding legacy sources of saltwater critical for managing stressed water resources along global coastlines.

## Abstract (web summary)

Hidden ancient saline groundwater beneath coastal Louisiana stems from a drowned Pleistocene valley and is revealed by large-scale airborne geophysical surveys.

## Introduction

**Fig. 1:**
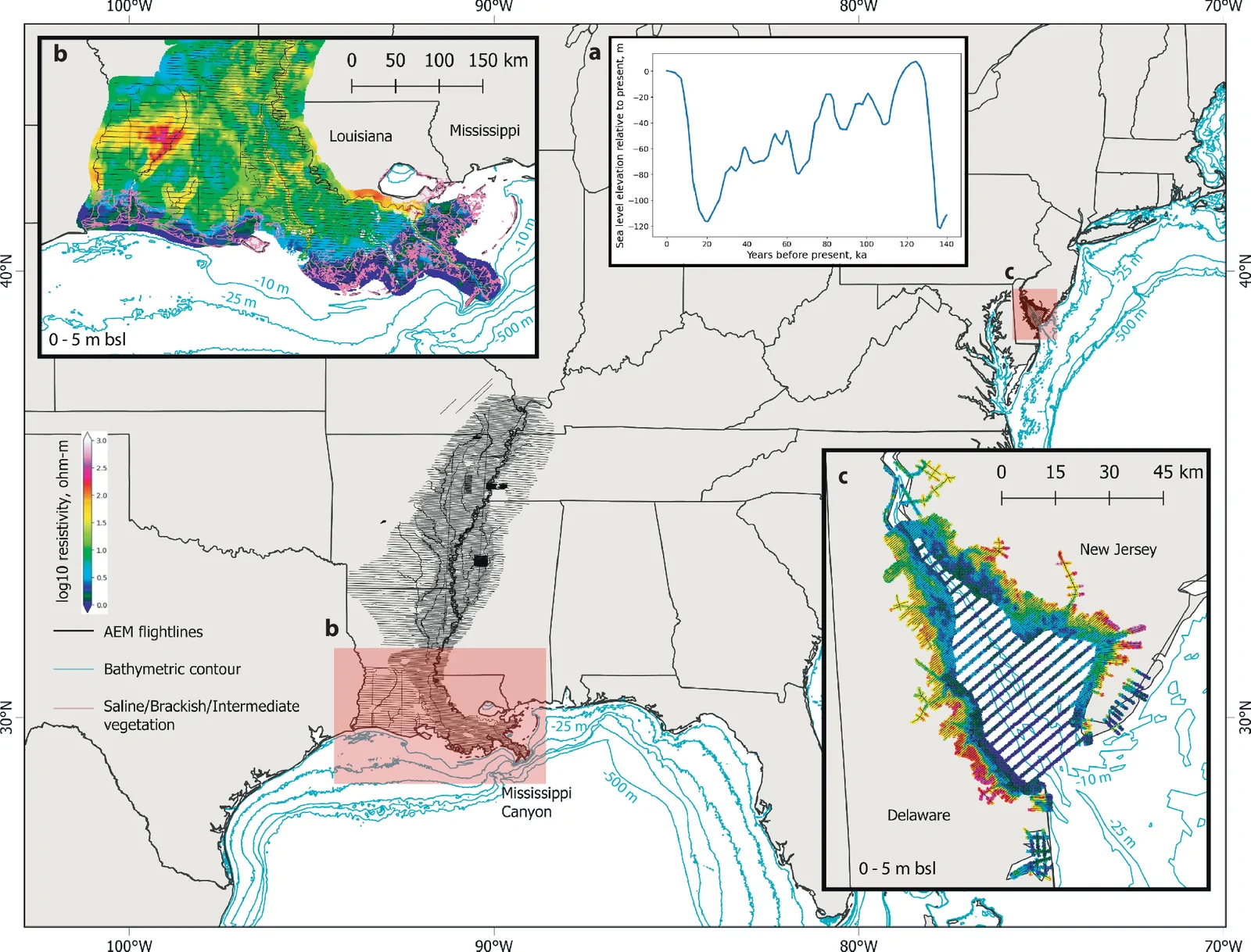
Summary of study areas in south-central and northeast United States. **a** Global mean sea level over the past 150 ka^65^. Map of AEM flightlines with inset of shallow electrical resistivity in the uppermost 0–5 m in the coastal Louisiana (**b**) and Delaware Bay (**c**) study areas. Blue contours show offshore bathymetry and pink contours in inset (**b**) indicate the extent of saltwater-impacted vegetation^31^.

Over 40% of Earth’s population lives within 100 km of coastlines^1,2^. Groundwater in coastal aquifers supports densely populated areas worldwide that rely on fresh water for municipal supply, agriculture, and industrial use, but is also vulnerable to contamination from saline water^2–6^. Saline and brackish groundwater occurs naturally in coastal regions, typically beneath a lens of freshwater that thickens inland. In the absence of other controlling factors, the position of the freshwater-saltwater interface is determined by the modern equilibrium between freshwater on land and sea level^7^. Saltwater intrusion, or inland movement of the interface, can be caused by natural and anthropogenic forces such as groundwater pumping, surface water management that alters natural recharge, rising sea levels in response to climate warming, and storm surges^3,4,7^. Geological processes have also altered the distribution of saline water in coastal regions globally, most notably since the last glacial maximum (LGM) approximately 20 ka, when global sea level was more than 120 m below present, which led to the formation of deeply incised river valleys as river systems attempt to reach equilibrium with a new lowered base level^8^. Rapid sea-level rise after the LGM (Fig. 1a) resulted in marine transgression and drowning of river valleys, filling of incised valleys with glacially and fluvially derived sediment, and in some locations formation of large delta systems that form the modern landscape. These infilled valleys can host permeable pathways that transmit seawater farther inland^9^, conduct fresh groundwater offshore^10^, or in some cases hold relict seawater from past marine incursions^11^, thus creating salinity distributions that are out of equilibrium with present-day conditions^12^. Knowledge of the preexisting distribution of saline groundwater and the hydrogeological structures that control its movement is therefore important for effective coastal groundwater resource management today, but is hindered by the lack of detailed subsurface data over large areas, and the process understanding needed to predict salinity.

Past studies have identified the presence of Holocene seawater in buried paleovalleys, supported by borehole data, modeling, geochemical observations, and geophysical surveys^11–15^. However, sparse point-sampling and ground-based geophysical observations leave significant gaps in the three-dimensional mapping of the subsurface that limit understanding of the past processes that created this saline groundwater legacy. Model-based studies can significantly underestimate the extent of inland groundwater salinity due to oversimplification of hydrogeologic properties or processes that are unknown or difficult to represent^6^, highlighting a need for advanced subsurface observational capabilities that can both identify locations of elevated salinity and develop understanding that enables its prediction in other settings. Here, we synthesize results of multiple large-scale, high-density airborne electromagnetic (AEM) surveys (Fig. 1b, c, “Methods”) to generate new system-scale understanding of the subsurface properties that reveal previously unseen details of coastal groundwater salinity and provide clues to their origins, with relevance to other Holocene incised valleys globally. These measurements are unprecedented in their scale and detail of subsurface mapping, and provide a new window into the uppermost several hundred meters belowground in three dimensions at catchment-to-basin scales that are critical for groundwater aquifer studies^16^.

This paper reports on new observations and insights into the legacy of ancient saline water recorded in the subsurface several hundred kilometers inland from the Louisiana coast. To support these findings, we leverage additional geophysical data from the present Delaware Bay drowned river valley estuary as a modern-day analog that yields insights into the origin of subsurface salinity in coastal Louisiana (Fig. 1). We compare the results of two separate AEM surveys (“Methods”) that contribute unparalleled data density for subsurface mapping over very large areas, with more than 3500 flight-line-kilometers of data covering about 3200 km^2^ surrounding Delaware Bay^17^, and more than 15,000 flight-line-kilometers covering over 64,000 km^2^ in southern Louisiana^18,19^ that is part of a larger basin-scale survey of the lower Mississippi River valley^16^. This latter survey represents the largest single AEM application for groundwater studies in the U.S. and demonstrates the capacity for remote subsurface mapping over very large areas. Characterization of the present-day salinity patterns identified beneath Delaware Bay, a modern drowned river valley estuary on the northeast Atlantic coastline of the United States (Fig. 1c), provides context for understanding the salinity distribution imaged within a large buried paleo-estuary beneath the Gulf Coast in southern Louisiana (Fig. 1b). This study highlights a space-for-time approach informed on multiple AEM studies links present and past subsurface properties and processes. Finally, we discuss how the saline water legacy identified by these data is out of equilibrium with modern conditions, impacting availability and future management of freshwater resources.

## Results and discussion

### A buried saline legacy beneath coastal Louisiana

The Mississippi River is North America’s largest drainage basin, forms one of the world’s largest deltaic plains, and is an important recorder of global environmental change^20^. Numerous studies have documented the Quaternary history of forces that have shaped the lower Mississippi River valley^21–27^, including discussion of a deeply incised valley during the LGM that discharged near the head of the Mississippi Canyon near the continental shelf^23,28^. Rapid sea-level rise approximately 15–10 ka led to a marine transgression that flooded the incised valley and formed a coastal embayment that is suggested to have reached about the latitude of Baton Rouge^23,29^. By 9–6 ka sea-level rise slowed then stabilized, when formation of an aggrading system of deltas began with the Maringouin Delta ~7.5 ka^21,23,29^. Water wells and vegetation types identify saline and brackish groundwater in the region, including some influence from dissolution of salt domes mostly in deeper parts of the aquifer system in parts of southwest Louisiana^30^; however, its large-scale distribution and relationship to the drowned river valley has not been detailed.

**Fig. 2:**
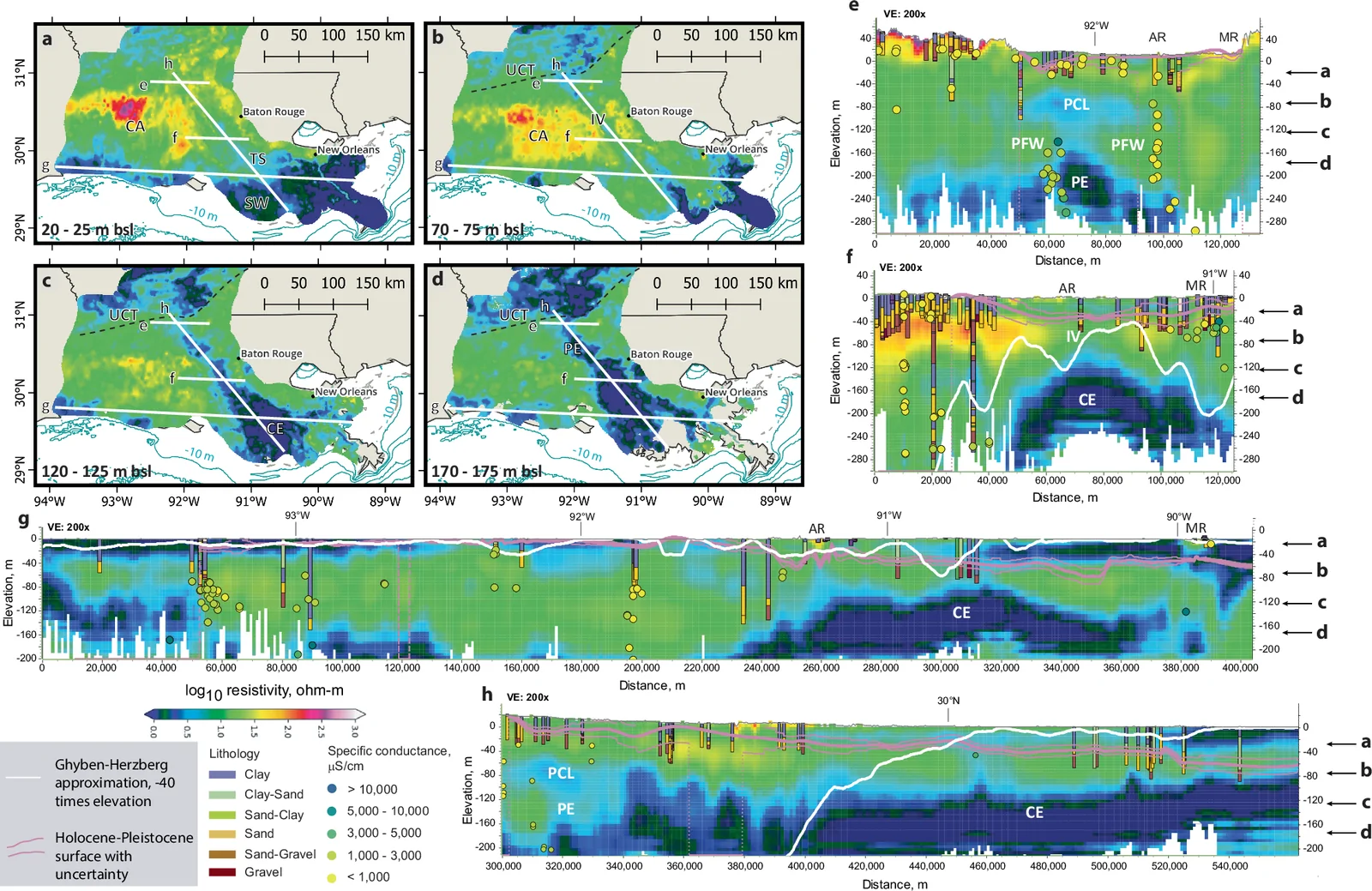
Coastal Louisiana resistivity models. Map-view slices of electrical resistivity at 20–25 m (**a**), 70–75 m (**b**), 120–125 m (**c**), and 170–175 m (**d**) elevation below sea level (bsl). Resistivity depth-sections along three west-east profiles from inland Louisiana (**e**) towards the Gulf Coast (**f**, **g**) and one northwest-southeast depth-section (**h**). Borehole lithology^34^ and specific conductance^35–37^ measurements are plotted on profiles, along with the GH-predicted base of fresh water (based on modern surface elevation). SW salt wedge, UCT upland-coastal transition, CA Chicot aquifer, TS topstratum, IV incised valley, CE coastal embayment, PE paleo-estuary, PCL Pleistocene clay, PFW paleo-fresh groundwater, MR Mississippi River, AR Atchafalaya River, VE vertical exaggeration.

AEM surveys acquired in 2021–2022 over the Gulf Coast region of Louisiana^18,19^ (“Methods”) extended previous investigations of regional groundwater availability in the lower Mississippi River valley^16^, and shed new light on the legacy of saline and brackish groundwater left by paleo-estuarine conditions that are now hidden beneath the modern deltaic plain. The shallowest resistivity models at 0–5 m below sea level (bsl) show prominent low resistivity (<3 ohm-m) near the Gulf Coast in southwest and southeast Louisiana (Fig. 1b) that correlate with areas of saltwater-impacted vegetation types^31^. At 20–25 m bsl (Fig. 2a), the low-resistivity saline wedge (SW) extends farther north as it dips beneath the coast. Geologic features evident at this depth are the more resistive (~10–300 ohm-m) Chicot aquifer system (CA) in southwest Louisiana and the moderately low-resistivity (~5–10 ohm-m) topstratum (TS) that comprises fine-grained clays that cap much of the deltaic plain and mark the Holocene-Pleistocene boundary (H-P) above coarser substratum that fills the bottom of deeply incised valleys^23,27,32^. The top of the incised valley (IV) is apparent by 70–75 m bsl (Fig. 2b) as a narrow low-resistivity (~5–25 ohm-m) band splitting the more resistive Chicot aquifer and Pleistocene uplands. The pattern of very low (<3 ohm-m) resistivity associated with the saline wedge at the Gulf Coast is largely replaced by intermediate resistivity (~5–25 ohm-m) at this depth, which we attribute to fresh groundwater that is likely discharged offshore.

Resistivity patterns shift significantly at greater depth, with a large funnel-shaped low-resistivity region (<3-4 ohm-m) extending as far north as Baton Rouge at 120 −125 m bsl (Fig. 2c). We interpret this feature as a saline relict of the aforementioned coastal embayment (CE) that was present as recently as ~12–6 ka^23^. Somewhat surprising is the extension of this low-resistivity feature at 170–175 m bsl (Fig. 2d) another ~75 km farther inland to the northwest, which we interpret as the subsurface legacy of a paleo-estuary (PE) formed by flooding of the incised valley. This low-resistivity feature continues to narrow inland until terminating in a channel emerging from Pleistocene and older units that mark a transition from upland alluvial to coastal plain regions (UCT, dashed line, Fig. 2b–d). This transition coincides with a change from the low-resistivity Pleistocene units farther north that have a steeper dip (~4–13 m/km) compared with the H-P surface (~0.2 m/km). This inferred paleo-estuary may have filled during the early stages of marine transgression^33^ before continued sedimentation during sea-level rise filled the coastal embayment (CE) that occupies a much larger area of nearly 10,000 km^2^. The smaller PE feature, around 1200 km^2^, is about 270 km from the nearest coastline (320 km from the nearby mouth of the Mississippi River), which is comparable to some of the farthest inland occurrences of paleo-saltwater in other Quaternary delta systems^11,13^.

Resistivity cross-sections together with borehole lithology^34^ and groundwater specific conductance^35–37^ observations (Fig. 2e–h) add spatial context to the map-view patterns. Moderately low resistivity in the near-surface correlates with the topstratum (TS) that forms a clay cap over much of the deltaic plain (Fig. 2e–h), overlying coarse-grained late Pleistocene substratum that filled the deeper portions of the incised valleys^32^. Shallow resistivity further decreases toward brackish and saline waters near the coast (Fig. 2g, h). While the presence of clays reduces electrical resistivity, values less than a few ohm-m are most strongly correlated with elevated groundwater salinity (Supplementary Discussion, Supplementary Table 1, Supplementary Fig. 1). The ambiguity in resistivity due to clay versus salinity is primarily relevant in fresh groundwater zones; at higher salinity the effect of clay on resistivity is reduced. Low resistivity is present towards the bottom of all four cross-sections, and is evident in map-view as the large funnel-shaped feature at depth (Fig. 2c, d), which we hypothesize is the fingerprint of a deep saline wedge left beneath the formerly drowned river valley.

Notably, the deep saline wedge does not occupy the incised valley itself but is found beneath it at depths greater than 120 m—it is the relict salinity of the groundwater flow system beneath the paleo-estuary and coastal embayment. This deep low-resistivity feature limits our ability to map the shape of the base of the incised valley because of the strong influence of salinity on resistivity, though the shape of the upper part of the valley is apparent as it bisects the more resistive Pleistocene uplands (IV in Fig. 2b). The deep saline wedge reflects the pattern of shallower H-P surface elevation contours^32^ (Supplementary Fig. 2), particularly south of Baton Rouge where the valley-shaped H-P interface indicates where Holocene marine transgression has left a deeper low-resistivity imprint. North of Baton Rouge the H-P contours corroborate a shift to the western edge of the alluvial valley coincident with locations of early Holocene meander belts of the Mississippi and Red Rivers^23^ that is also captured in detail in the low-resistivity feature marked as PE (Fig. 2d and Supplementary Fig. 2). However, far fewer interpolation points of the H-P surface^32^ do not capture the detailed geometry of the region interpreted as a paleo-estuary as clearly shown by the resistivity maps, further demonstrating the value of spatially extensive geophysical data to capture features under-sampled by sparse or irregular ground-based observations.

In the area described as marking a paleo-estuary (Fig. 2d), there is also a moderately low-resistivity halo sitting above the deep low-resistivity evident in cross-section view (PCL in Fig. 2e), which corresponds with a region of elevated shallow groundwater salinity^38^. Supported by borehole observations at the bottom of several wells in this location^34^ and its position beneath the H-P surface^32^, we hypothesize that this low-resistivity halo represents late Pleistocene clays and other more consolidated sediments. The shallow low-resistivity halo and deeper salt wedge is flanked by moderate resistivity (PFW in Fig. 2e), which we conjecture represents paleo-fresh groundwater (PFW) surrounding the saline wedge that is now preserved in the subsurface. This shallow low-resistivity halo is only evident above the northernmost parts of the region marked by PE in Fig. 2d; south of this location, it may either have never existed or have been eroded by major floods and channel-filling with coarse-grained substratum sediments^26,29^. It is also similar to other findings of trapped seawater in low-permeability valley-filling sediments^11^; however, this feature is far less prominent and extensive than the deeper low-resistivity associated with the saltwater wedge that formed and is now preserved beneath the incised valley.

The white curves shown on Fig. 2f–h illustrate the theoretical depth to the base of fresh water predicted by the Ghyben-Herzberg (GH) sharp-interface approximation^39^ based on the elevation of the water table (approximated as land-surface elevation in this low-lying region) and the relative density of fresh and saline groundwater. Unlike with the modern Delaware Bay system discussed in the next section, the GH-predicted depth to the salt-water wedge (white curves, Fig. 2f–h) for present-day hydrologic conditions does not follow the deep low-resistivity feature here because the saline groundwater was emplaced under paleo-topography and hydrology that no longer exist. In the northernmost cross-section (Fig. 2e), the modern land surface is about 15 m above sea level and, with relatively shallow depth to the water table (~5–7 m), the GH-predicted elevation is well beneath the displayed profile. Equilibration with the modern land surface and water table elevation would cause the depth of the salt-water wedge to increase over time. Saline wedges are seen dipping from the near surface near coastlines, for example in the southwest corner of Louisiana (left side of Fig. 2g), southeast of New Orleans near the edge of the St. Bernard delta lobe (right side of Fig. 2g), and along the southern coastline at the end of the valley-parallel profile (Fig. 2h).

### Comparison with Delaware Bay: a modern drowned river valley estuary

We next discuss the similarities found between the buried saline features discussed above from coastal Louisiana and Delaware Bay, situated in a modern drowned river valley. Delaware Bay is a modern tidal estuary between Delaware and New Jersey, occupying an area of about 1700 km^2^ at the terminus of the Delaware River before it reaches the Atlantic Ocean (Fig. 1c). The bay at present-day sea level fills a drowned river valley formed during the LGM, with a maximum water depth of about 45 m. Aquifers adjacent to Delaware Bay provide a fresh water supply for the densely populated coastal communities, and are in some cases experiencing active saltwater intrusion^40,41^. An AEM survey was conducted over Delaware Bay and surrounding regions of Delaware and New Jersey in 2022^17^ (“Methods”) to improve baseline characterization of the freshwater-saltwater interface that can be used to inform studies of saltwater intrusion^42^ and effective water management.

**Fig. 3:**
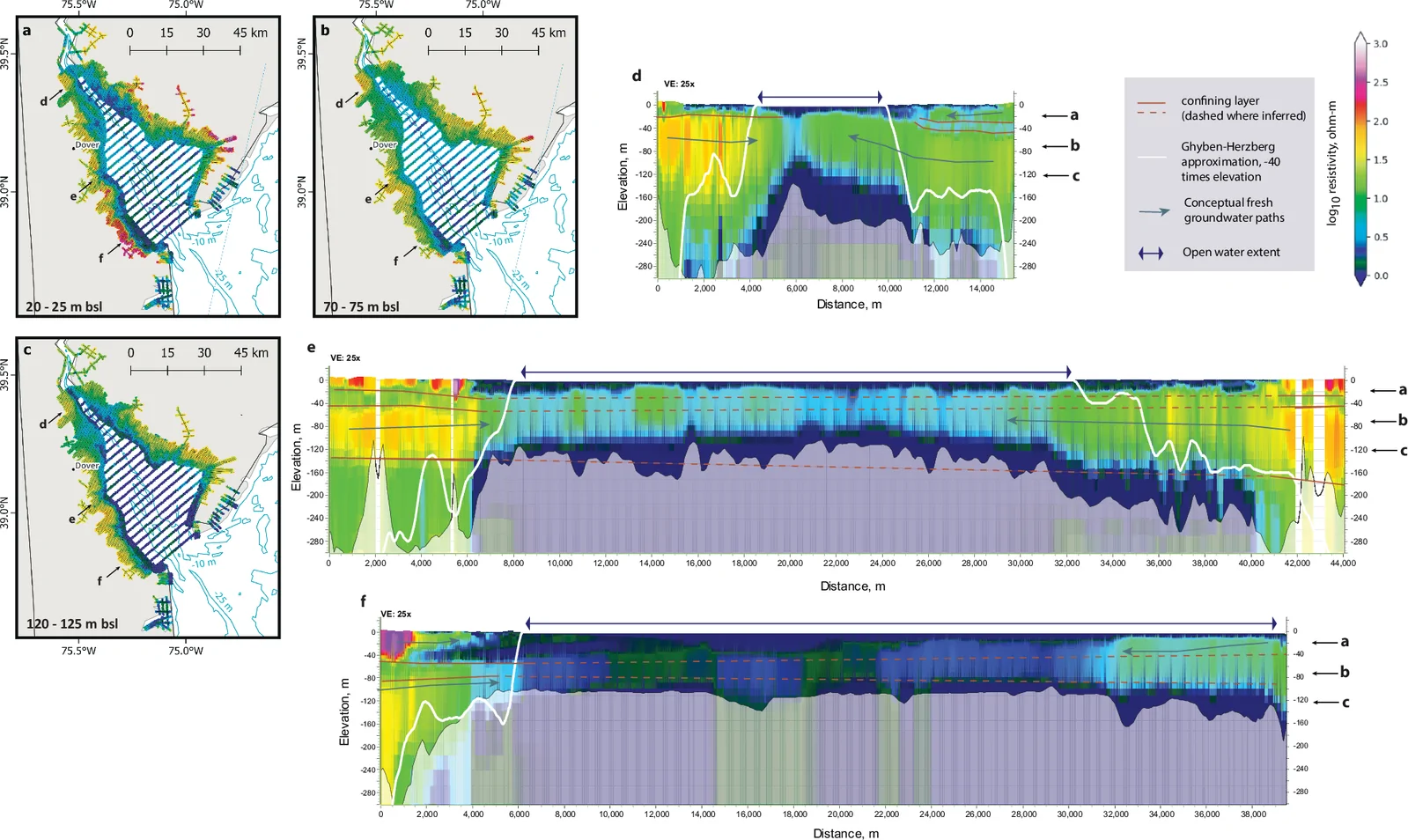
Delaware Bay resistivity models. Map-view slices of electrical resistivity at 20–25 m (**a**), 70–75 m (**b**), and 120–125 m (**c**) elevation below sea level (bsl). Resistivity depth-sections along three profiles near the head (**d**), middle (**e**), and mouth (**f**) of Delaware Bay. White transparency shading towards the bottom of each profile indicates the depth of investigation where resistivity values are not well-constrained by data. VE vertical exaggeration.

Low electrical resistivity, indicative of elevated salinity, is evident in map view at shallow depths beneath Delaware Bay and surrounding wetland areas (Figs. 1c and 3a). Low shallow resistivity is also evident beneath Delaware Bay in cross-section views (Fig. 3d–f). The areas of highest salinity relate to the lowest resistivity values (dark blue colors) ~0.3–3 ohm-m, with seawater at the lower end of that range and resistivity increasing with decreasing salinity. Landward of the bay towards Delaware and New Jersey, a stacked sequence of aquifers (higher resistivity, typically above ~25 ohm-m) and confining layers (lower resistivity, ~5–25 ohm-m) are evident, with intermediate resistivity at mid-depths beneath the low-resistivity bay (Fig. 3b, d–f) that we attribute to mixing with discharging fresh groundwater. At greater depth (Fig. 3c, d–f), low resistivity is again prevalent beneath the footprint of Delaware Bay, dipping more steeply to greater depths landward of the bay. We attribute this deep low-resistivity feature to the saline groundwater wedge that exists beneath surface seawater bodies.

In cross-section view (Fig. 3 d–f and Supplementary Fig. 3), resistivity data show saline groundwater extending landward of the footprint of the Delaware Bay with a distribution typical of sandy coastal aquifers. A thin surficial zone of elevated salinity extends inland, reflecting tidal influence across coastal wetlands. Beneath the surface salinity is a freshwater body that terminates at a freshwater-saltwater interface deepening landward, indicating freshwater discharging toward the bay above a typical saltwater wedge. Here, unlike in coastal Louisiana, the pattern predicted by GH (Fig. 3d–f and Supplementary Fig. 3) closely reflects the pattern of deep low resistivity observed from the AEM data outside the footprint of Delaware Bay, supporting our interpretation of this feature as the base of freshwater above a saltwater wedge. Beneath the bay, freshened groundwater at mid-depths separates the low-resistivity intervals observed in the near surface and the deep saline groundwater at depth. The freshened groundwater beneath the bay is likely a dispersed mixing zone between the saltwater in the bay and freshwater discharged through heterogeneous aquifer and confining strata extending beneath the bay^42^.

The spatial patterns of salinity interpreted from AEM-derived resistivity data surrounding Delaware Bay share many similarities with observations in coastal Louisiana, allowing for an illustrative comparison despite differences in scale, sediment supply, tectonic setting, and climate history that may also be relevant in other coastal Holocene incised valleys globally. The deep saltwater wedge beneath the modern Delaware Bay incised valley (~1700 km^2^) represents subsurface saline conditions that would have existed in coastal Louisiana after drowning of the incised river valley over the similar-sized (~1200 km^2^) paleo-estuary (PE in Fig. 2d) and then the much-larger (~10,000 km^2^) coastal embayment (CE in Fig. 2c), which are now preserved in the subsurface. Additionally, the preserved paleo-freshwater near the head of the buried paleo-estuary (PFW in Fig. 2e) closely mimics the pattern of freshened groundwater discharging beneath Delaware Bay today (Fig. 3d–e). Together, these results support our interpretations that the saltwater wedge and adjacent freshwater discharge from the ancient paleo-estuary in coastal Louisiana are preserved beneath the modern deltaic plain, but out of equilibrium with current hydrologic conditions.

### Past geological processes leave a groundwater salinity legacy

**Fig. 4:**
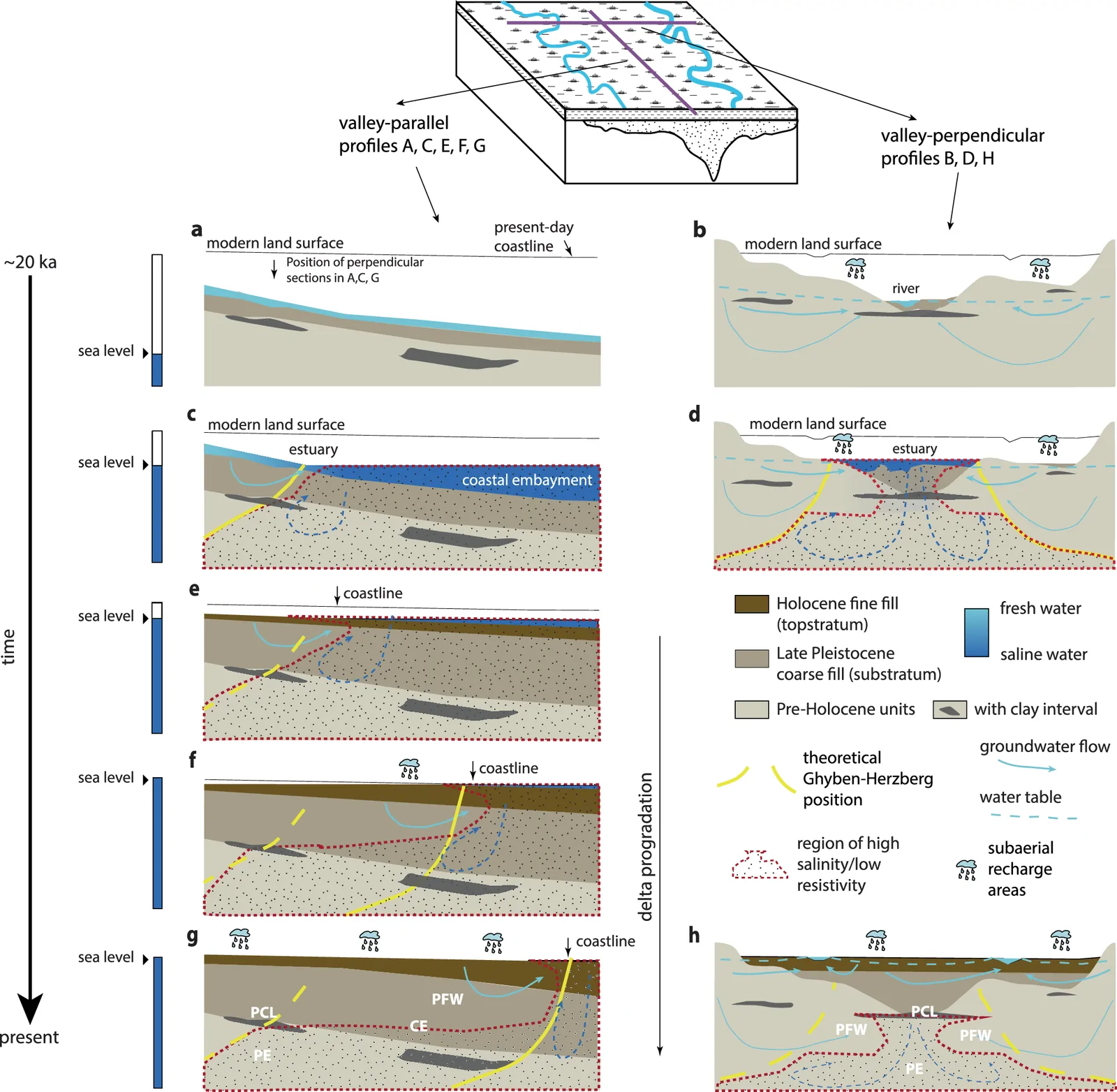
Summary of the saline groundwater legacy left by a buried paleo-estuary. A deeply incised valley (**a**, **b**) is drowned after sea level rise (**c**, **d**), forming a shallow estuary with a salt wedge in the subsurface. The theoretical sharp Ghyben-Herzberg (GH) interface is shown as yellow lines (dashed at its original position at maximum transgression), and the stippled region enclosed by red dashed lines shows the area of elevated salinity. Further post-glacial sediment fills in the incised valley and buries it beneath floodplain and prograding delta deposits (**e**–**h**).

This study describes a sequence of incised valley formation during periods of sea-level lowstands (Fig. 4a, b) followed by sea-level rise and marine transgression that forms a shallow inland tidal estuary (Fig. 4c, d) such as the modern Delaware Bay or late Pleistocene Gulf Coast. Saline and brackish water at the surface extends into the subsurface as a saltwater wedge that deepens landward and mixes with fresh groundwater inputs beneath the estuary (Figs. 4c, d and 3d–f). Further sediment deposition that exceeds accommodation space can bury the paleo-incised valley and result in an aggrading delta system that buries a legacy of hidden saline and brackish water in the subsurface (Figs. 4e–g and 2e–h). Saltwater circulation is expected to abate after burial of the paleo-estuary and removal of saline surface water that creates strong salinity gradients.

Model-based studies have shown that saline water can be trapped and preserved beneath prograding delta systems over large distances and timescales in excess of 16,000 years for scenarios consistent with coastal Louisiana, with rapid progradation during the Holocene and heterogeneous confining layers at depth^43^. Near-surface saline water introduced during Holocene transgressions can rapidly infiltrate downwards through coarse-grained incised valley fill such as the late Pleistocene substratum, slowing where confining layers or the less permeable base of valleys is encountered^44^. Freshening of the upper substratum and Holocene topstratum occurs during progradation, leaving the saltwater legacy at depth beneath a shallow freshwater system^43,44^. Present-day hydrologic conditions would tend to flush the saline water out of the system, but the rate of basin-scale flushing decreases exponentially with depth^45^, particularly in deltaic systems with characteristic heterogeneity that creates high vertical anisotropy in permeability^46,47^. Thus, the combined effects of low-permeability topstratum acting as a barrier to recharge of freshwater, deeper low-permeability layers, and the slow rate of deep groundwater flow in low hydraulic gradient coastal regions allow the saltwater to persist over glacial timescales^44–48^.

**Fig. 5:**
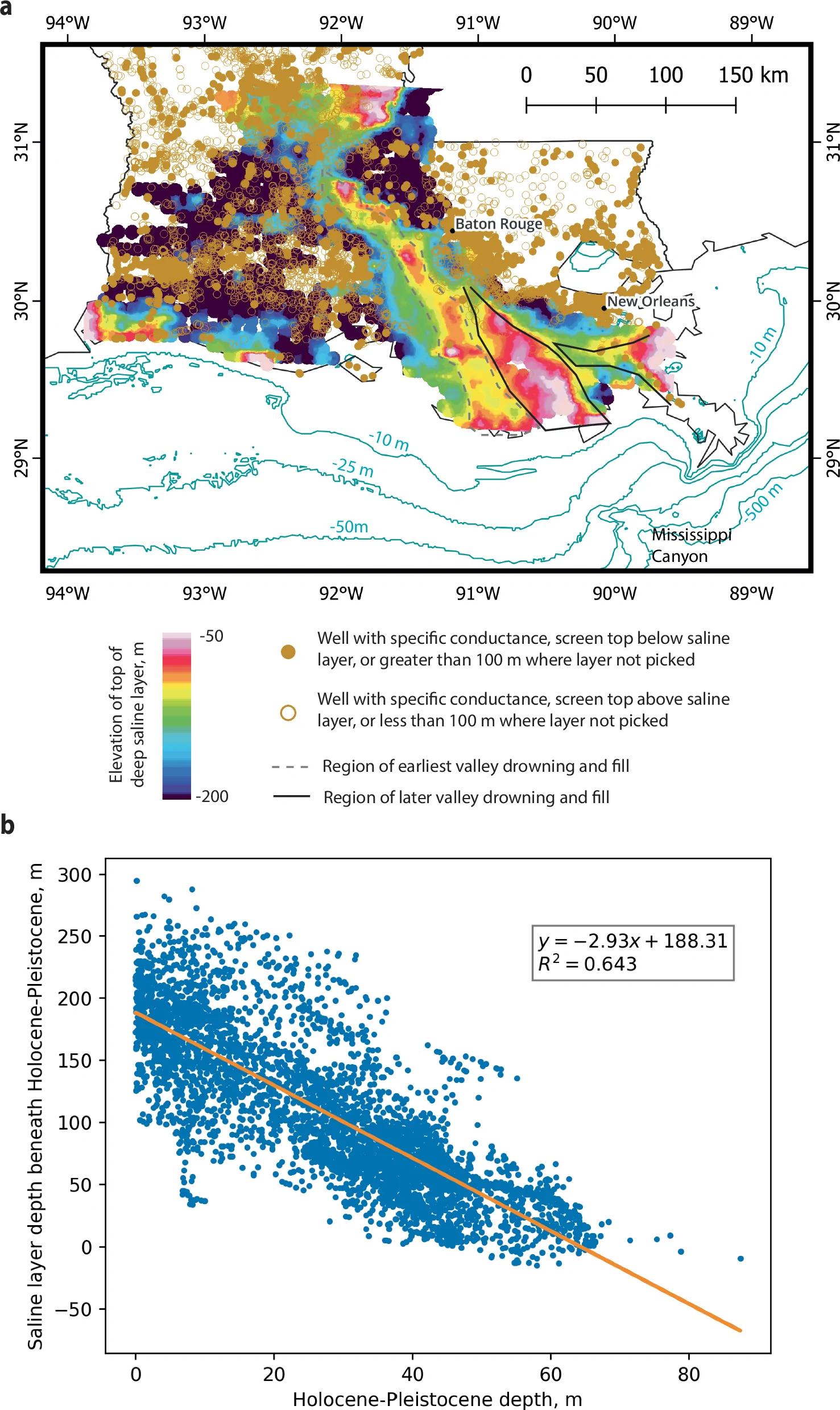
Summary of the saline groundwater legacy left by a buried paleo-estuary. **a** The interpreted top-elevation of the buried saline groundwater layer shows the extent and spatial patterns of past valley inundation and filling and corresponds to a region generally absent of water wells. **b** Relationship between the depth of the Holocene-Pleistocene contact^32^ (Supplementary Fig. 2) and depth to the top of the saline layer in (**a**).

We summarize this buried region of elevated groundwater salinity in coastal Louisiana by mapping the top elevation of the low-resistivity feature (<3−4 ohm-m), which highlights multiple distinct regions of elevated salinity related to the saline wedge left beneath previously inundated valleys (Fig. 5a). A lower-elevation channel (grey-dashed polygon) outlines what may be the main axis of the incised valley that was drowned first as sea levels rose and is distinct from shallower salt wedges to the east that shallow to the Gulf Coast and reflect a modern connection to saline surface water (solid black polygons). The presence of this buried saltwater legacy is partly evident from the lack of water wells over this feature (Fig. 5a) and highlights its strong effect on modern water supply infrastructure. There are very few wells that overlap with the extent of the buried saltwater feature, and most of the wells that exist are not as deep as the top of the conductive layer (shown as open circles); thus, they are tapping the shallower fresh groundwater. The position of the top of the saline layer (Fig. 5a) relative to the base of the H-P interface (Supplementary Fig. 2) is correlated (*R*^2^ = 0.643) with the depth of the H-P interface (Fig. 5b), further suggesting that drowning of Holocene valleys is the cause of the modern high salinity region at depth. That is, elevated salinity at depths that would otherwise contain freshwater is observed beneath the location of Holocene valleys, as that saltwater equilibrates with modern hydrologic conditions.

While this provides further evidence for the presence of non-potable saline water at depth, the AEM results stand out in their utility as a management tool by identifying the regions of elevated groundwater salinity that could be vulnerable to saltwater intrusion if over-pumped. Conversely, deep fresh water that exists beneath the theoretical GH interface is tapped by wells and is also a relict of the paleo-freshwater flow system. Supplementary Fig. 4 shows resistivity data with sampled groundwater ages^49^ and salinity^35–37^. Groundwater age sample locations are compared with the theoretical GH position, and locations with low salinity that are near-to-below the GH curve indicate wells that may be intersecting relict fresh water. Thus, understanding the geologic history of coastal aquifers and its impact on the modern-day distribution of groundwater salinity, which may be out of equilibrium with current hydrologic conditions, is an important component of effective water resource management in populated coastal regions globally^11,12,15^. Our approach of regional-scale subsurface mapping to identify modern groundwater salinity patterns that reflect past geological processes is likely relevant to other major delta systems globally given the prevalence of late-Quaternary incised valleys^50^ and the worldwide formation of marine deltas during Holocene sea level rise^51^.

## Methods

Our study synthesizes results from two large AEM studies conducted over coastal regions—coastal Louisiana and areas in Delaware and New Jersey surrounding Delaware Bay—to improve understanding of regional-scale subsurface salinity distributions and hydrogeologic properties that are difficult to obtain using ground-based observations. AEM data effectively fill a scale gap in subsurface imaging that bridges other established approaches like lidar or Landsat for understanding Earth’s surface properties, or GRACE satellite data for mapping global changes in subsurface water storage^52^. Growing use of AEM data has revealed buried paleovalley structures^53–55^ and groundwater salinity patterns^56–58^, demonstrating the value of remote mapping of large-scale subsurface structures that cannot be achieved otherwise. All components of the AEM lifecycle from conceptualization and planning through data processing and model integration were conducted by the USGS and co-authors, except for data acquisition that was carried out through contracts awarded to commercial geophysical companies with expertise in AEM surveys. Our analysis relies primarily on interpretation of the AEM results, together with compilation of existing information about subsurface lithology and salinity from borehole observations.

### Airborne electromagnetic surveys

Extensive AEM data were acquired over southern Louisiana (Fig. 1b) as part of a larger survey of the lower Mississippi River valley from September 2021–January 2022, and that extended several previous phases of AEM acquisition in the region^16^. Data were acquired with the Xcalibur Tempest system, a time-domain electromagnetic instrument together with a CS-3 cesium vapor magnetometer and a Radiation Solutions RS-500 gamma-ray spectrometer deployed by a Cessna 208B fixed-wing aircraft. This subset of the larger regional survey comprised 27,204 line-kilometers flown at a nominal flight height of 120 m above terrain, primarily along parallel flight-lines oriented west–east and with nominal line spacing of 3–6 km. Several irregular flightlines were acquired along the Mississippi and Atchafalaya Rivers. Survey details and the contractor report are publicly available^18^. About 16,000 line-kilometers of this survey are within the area presented in this study.

A second, smaller AEM survey was acquired over several rivers in the lower Mississippi River valley, including several rivers in southwestern Louisiana included in this study^59^, from May–August 2021. Data were acquired with the Xcalibur Resolve system, a frequency-domain electromagnetic instrument together with a CS-3 cesium vapor magnetometer and a Radiation Solutions RS-500 gamma-ray spectrometer deployed by a Eurocopter AStar 350 B3 helicopter. The survey comprised 10,706 line-kilometers flown at a nominal flight height of 30–40 m above terrain, primarily along irregular paths that followed river and levee systems in the lower Mississippi River valley and Chicot aquifer systems. Survey details and the contractor report are publicly available^59^. About 1800 line-kilometers of this survey are within the area presented in this study

AEM data were acquired over Delaware Bay and surrounding parts of Delaware and New Jersey (Fig. 1c) from July–August 2022. Data were acquired with the SkyTEM 304 M time-domain electromagnetic system together with a Geometrics G822A cesium vapor magnetometer slung beneath a Eurocopter AStar 350 B3 helicopter. The survey comprised 3588.5 line-kilometers flown at a nominal flight height of 30–40 m above terrain along parallel flightlines primarily oriented southwest-northeast and with nominal line spacing of 500 m over land and nearshore, and 3 km over open-water parts of Delaware Bay. Several irregular flightlines were acquired along, and perpendicular to, nearby rivers to capture salinity patterns along these inland corridors of likely groundwater-surface water interaction. Survey details and the contractor report are publicly available^17^.

### Processing and inversion of airborne electromagnetic data

Tempest data from coastal Louisiana were processed using Python scripts to remove interference from man-made infrastructure such as powerlines and buildings and averaged to common sounding locations to reduce random noise. Data were inverted using the GALEISBSTDEM 1-D time-domain deterministic inversion software^60^ (Geoscience Australia, Release-20160606). Inversions were done using a multilayered smooth model formulation in which resistivity values were recovered for 30 layers that increase in thickness from the surface to 300 m depth. Resistivity models were calculated at over 450,000 locations, though only about one-third of those are in the coastal Louisiana study area presented here. Estimates of the depth of investigation (DOI), or depth to which models are constrained by observations for each location, were calculated using multiple inversions with different reference models following the approach of Oldenburg and Li^61^. Data and inverted models, along with acquisition and processing information, are publicly available^18^.

Resolve data from coastal Louisiana were imported into the Aarhus Workbench software (Aarhus GeoSoftware, ver. 6.1.0.0,) where manual data edits were made to remove interference from man-made infrastructure such as powerlines and buildings and averaged to common sounding locations to reduce random noise. Data were inverted in Aarhus Workbench using a one-dimensional laterally constrained (1DLCI) inversion that solves for the electrical resistivity in each of 30 layers that increase in thickness from the surface to 125 m depth. Resistivity models were calculated at over 748,000 locations, though only a small fraction of which are in the southwest Louisiana study presented here, with estimates of the DOI based on the approach of Christiansen and Auken^62^. Data and inverted models, along with acquisition and processing information, are publicly available^59^.

SkyTEM data from Delaware Bay were imported into the Aarhus Workbench software (Aarhus GeoSoftware, ver. 6.6–2024.1), where manual data edits were made to remove interference from man-made infrastructure such as powerlines and buildings and averaged to common sounding locations to reduce random noise. Data were inverted in Aarhus Workbench using a one-dimensional laterally constrained (1DLCI) inversion that solves for the electrical resistivity in each of 40 layers that increase in thickness from the surface to 300 m depth. Resistivity models were calculated at 78,684 locations, with estimates of the DOI based on the approach of Christiansen and Auken^62^. Data and inverted models, along with acquisition and processing information, are publicly available^17^.

### Developing a combined 3D resistivity model for coastal Louisiana

Inverted resistivity models from the Tempest and Resolve AEM surveys, together with data from previous AEM surveys in the lower Mississippi River valley, were merged into a regular three-dimensional resistivity grid aligned to the 1 km-resolution national hydrogeologic grid^63^ with 5 m vertical discretization. Resistivity models that fall within a 1 km × 1 km × 5 m grid cell were averaged according to their respective vertical lengths from the 1-D models that intersected that cell. Averaged Tempest and Resolve models were separately interpolated using kriging via the GeostatsPy package (version 0.0.19, https://pypi.org/project/geostatspy/) and the GSLIB Geostatistical Software Library^64^. The kriged grids were combined using a weighted function that smoothly transitioned from a mix of 0.52/0.48 Resolve/Tempest in the shallowest layer, to 0.00/1.00 Resolve/Tempest by a depth of 75 m (typical of the shallower Resolve DOI compared with Tempest), to produce the final resistivity models presented here (Fig. 2)^19^. This approach for blending resistivity models for the two systems was developed farther north in the lower Mississippi River valley where interleaving Tempest and Resolve flights were common^16^. However, in the study area presented here, about 90% of the nearly 18,000 line-kilometers were flown with Tempest. The Resolve system was only flown along some of the rivers in southwest Louisiana, and along part of the Mississippi River upstream of New Orleans, and therefore does not significantly impact the merged resistivity grids presented here.

## Supplementary information

Supplementary Information

Transparent Peer Review File

## Data Availability

The AEM datasets and inversion results presented in this study are available online in the USGS ScienceBase repository: Delaware Bay (10.5066/F7J96592). Louisiana (10.5066/P9KPK3UJ, 10.5066/P93DO0EO, 10.5066/P9382RCI).

## References

[1] Lansu, E. M. et al. A global analysis of how human infrastructure squeezes sandy coasts. *Nat. Commun.* **15**, 432 (2024). 38199992 10.1038/s41467-023-44659-0 PMC10781753

[2] Richardson, C. M. et al. The impacts of climate change on coastal groundwater. *Nat. Rev. Earth Environ.* **5**, 100–119 (2024).

[3] Jasechko, S., Perrone, D., Seybold, H., Fan, Y. & Kirchner, J. W. Groundwater level observations in 250,000 coastal US wells reveal scope of potential seawater intrusion. *Nat. Commun.* **11**, 3229 (2020). 32591535 10.1038/s41467-020-17038-2 PMC7319989

[4] Michael, H. A., Post, V. E. A., Wilson, A. M. & Werner, A. D. Science, society, and the coastal groundwater squeeze. *Water Resour. Res.* **53**, 2610–2617 (2017).

[5] Gleeson, T. et al. Groundwater sustainability strategies. *Nat. Geosci.* **3**, 378–379 (2010).

[6] Zamrsky, D., Oude Essink, G. H. P. & Bierkens, M. F. P. Global impact of sea level rise on coastal fresh groundwater resources. *Earth’s Future* **12**, e2023EF003581 (2024).

[7] Werner, A. D. et al. Seawater intrusion processes, investigation and management: recent advances and future challenges. *Adv. Water Resour.* **51**, 3–26 (2013).

[8] Blum, M. D. & Törnqvist, T. E. Fluvial responses to climate and sea-level change: a review and look forward. *Sedimentology* **47**, 2–48 (2000).

[9] Yu, X. & Michael, H. A. Mechanisms, configuration typology, and vulnerability of pumping-induced seawater intrusion in heterogeneous aquifers. *Adv. Water Resour.* **128**, 117–128 (2019).

[10] Russoniello, C. J. et al. Geologic effects on groundwater salinity and discharge into an estuary. *J. Hydrol.* **498**, 1–12 (2013).

[11] Larsen, F. et al. Groundwater salinity influenced by Holocene seawater trapped in incised valleys in the Red River delta plain. *Nat. Geosci.* **10**, 376–381 (2017).

[12] Sheng, C. et al. Evolution of groundwater system in the Pearl River Delta and its adjacent shelf since the late Pleistocene. *Sci. Adv.* **10**, eadn3924 (2024). 38598633 10.1126/sciadv.adn3924 PMC11006231

[13] Rahman, M., Tokunaga, T. & Yamanaka, T. Origin and evolutionary processes of deep groundwater salinity in southwestern coastal region of the Ganges-Brahmaputra-Meghna Delta, Bangladesh. *J. Hydrol. Reg. Stud.* **36**, 100854 (2021).

[14] Meyer, R., Engesgaard, P. & Sonnenborg, T. O. Origin and dynamics of saltwater intrusion in a regional aquifer: combining 3-d saltwater modeling with geophysical and geochemical data. *Water Resour. Res.* **55**, 1792–1813 (2019).

[15] Le, H. et al. Buried deep freshwater reserves beneath salinity-stressed coastal Bangladesh. *Nat. Commun.* **16**, 10740 (2025). 41315357 10.1038/s41467-025-65770-4 PMC12663281

[16] Minsley, B. J. et al. Airborne geophysical surveys of the lower Mississippi Valley demonstrate system-scale mapping of subsurface architecture. *Commun. Earth Environ.* **2**, 131 (2021).

[17] U.S. Geological Survey. Airborne electromagnetic and magnetic survey of Delaware Bay and surrounding regions of New Jersey and Delaware, 2022. *U.S. Geological Survey data release*. 10.5066/F7J96592.

[18] Hoogenboom, B. E., Minsley, B. J., James, S. R. & Pace, M. D. Airborne electromagnetic, magnetic, and radiometric survey of the Mississippi Alluvial Plain, Mississippi Embayment, and Gulf Coastal Plain, September 2021 - January 2022: *U.S. Geological Survey data release*. 10.5066/P93DO0EO (2023).

[19] James, S. R. & Minsley, B. J. Combined results and derivative products of hydrogeologic structure and properties from airborne electromagnetic surveys in the Mississippi Alluvial Plain (ver. 2.0, July 2024). *U.S. Geological Survey data release*. 10.5066/P9382RCI (2021).

[20] Bianchi, T. S. & Allison, M. A. Large-river delta-front estuaries as natural “recorders” of global environmental change. *Proc. Natl. Acad. Sci. USA* **106**, 8085–8092 (2009). 19435849 10.1073/pnas.0812878106 PMC2688901

[21] Fisk, H. N. *Geological Investigation of the Alluvial Valley of the Lower Mississippi River*. 78 (Mississippi River Commission, 1944).

[22] Autin, W. J., Burns, S. F., Miller, B. J., Saucier, R. T. & Snead, J. I. Quaternary geology of the Lower Mississippi Valley. in *Quaternary Nonglacial Geology* (ed. Morrison, R. B.), vol. K.-2 0 (Geological Society of America, 1991).

[23] Saucier, R. T. *Geomorphology and Quaternary Geologic History of the Lower Mississippi Valley*. (U.S. Army Engineer Waterways Experiment Station, 1994).

[24] Blum, M. D. & Roberts, H. H. Drowning of the Mississippi Delta due to insufficient sediment supply and global sea-level rise. *Nat. Geosci.* **2**, 488–491 (2009).

[25] Blum, M. D., Tomkin, J. H., Purcell, A. & Lancaster, R. R. Ups and downs of the Mississippi Delta. *Geology* **36**, 675–678 (2008).

[26] Rittenour, T. M., Blum, M. D. & Goble, R. J. Fluvial evolution of the lower Mississippi River valley during the last 100 k.y. glacial cycle: response to glaciation and sea-level change. *Geol. Soc. Am. Bull.* **119**, 586 (2007).

[27] Kulp, M. et al. Latest quaternary stratigraphic framework of the Mississippi River delta region. *Gulf Coast Assoc. Geol. Soc.Trans.* **573**, 582 (2002).

[28] Wessels, S. *Late Quaternary Mississippi River Incised Valley Fill: Transgressive Depositional Packages* (University of New Orleans, 2010).

[29] Bentley, S. J., Blum, M. D., Maloney, J., Pond, L. & Paulsell, R. The Mississippi River source-to-sink system: perspectives on tectonic, climatic, and anthropogenic influences, Miocene to Anthropocene. *Earth Sci. Rev.* **153**, 139–174 (2016).

[30] Williamson, A. K. & Grubb, H. F. Ground-water flow in the Gulf Coast aquifer systems, south-central United States. *U.S. Geological Survey Professional Paper 1416-F*. 10.3133/pp1416F (2001).

[31] Nyman, J. A. et al. Vegetation types in coastal Louisiana in 2021 (ver. 2.0, April 2023). *U.S. Geological Survey data release.*10.5066/P9URYLMS (2022).

[32] Heinrich, P., Paulsell, R., Milner, R., Snead, J. & Peele, H. *Investigation and GIS Development of the Buried Holocene-Pleistocene Surface in the Louisiana Coastal Plain* (Louisiana Geological Survey-Louisiana State University for Coastal Protection and Restoration Authority of Louisiana, Baton Rouge, LA, 2015).

[33] Kesel, R. H. A revised holocene geochronology for the lower Mississippi valley. *Geomorphology* **101**, 78–89 (2008).

[34] U.S. Geological Survey, Louisiana Department of Natural Resources, Louisiana Water Resources Research Institute, Tsai, F. & Elkharankany, M. National Water-Well Database (NWWDB), Louisiana. *U.S. Geological Survey data release.*10.5066/P137JNNH (2026).

[35] Blondes, M. S. et al. U.S. Geological Survey National Produced Waters Geochemical Database (ver. 3.0, December 2023). *U.S. Geological Survey data release*. 10.5066/P9DSRCZJ (2023).

[36] Qi, S. & Harris, A. C. Geochemical Database for the Brackish Groundwater Assessment of the United States. *U.S. Geological Survey data release*. 10.5066/F72F7KK1 (2017).

[37] National Water Quality Monitoring Council, United States Geological Survey (USGS), & Environmental Protection Agency (EPA). Water Quality Portal. 10.5066/P9QRKUVJ (2021).

[38] Smoot, C. W. Louisiana hydrologic atlas map no. 3: altitude of the base of freshwater in Louisiana. *U.S. Geological Survey Water-Resources Investigations Report 86-4314*. 10.3133/wri864314 (1988).

[39] Post, V. E. A., Houben, G. J. & van Engelen, J. What is the Ghijben-Herzberg principle and who formulated it? *Hydrogeol. J.* **26**, 1801–1807 (2018).

[40] Panthi, J., Pradhanang, S. M., Nolte, A. & Boving, T. B. Saltwater intrusion into coastal aquifers in the contiguous United States — A systematic review of investigation approaches and monitoring networks. *Sci. Total Environ.* **836**, 155641 (2022). 35513146 10.1016/j.scitotenv.2022.155641

[41] Barlow, P. M. & Reichard, E. G. Saltwater intrusion in coastal regions of North America. *Hydrogeol. J.* **18**, 247–260 (2010).

[42] Hingst, M. C. et al. Beyond the wedge: impact of tidal streams on salinization of groundwater in a coastal aquifer stressed by pumping and sea-level rise. *Water Resour. Res.* **60**, e2023WR035840 (2024).

[43] Voller, V. R. et al. Onshore entrapment of seawater in coastal aquifers by rapid coastline progradation. *Water Resour. Res.* **61**, e2024WR038157 (2025).

[44] Delsman, J. R. et al. Paleo-modeling of coastal saltwater intrusion during the Holocene: an application to the Netherlands. *Hydrol. Earth Syst. Sci.* **18**, 3891–3905 (2014).

[45] Tóth, J. The evolutionary concepts and practical utilization of the tóthian theory of regional groundwater flow. *Int. J. Earth Environ. Sci.* **2016**, 10.15344/2456-351X/2016/111 (2016).

[46] Michael, H. A. & Voss, C. I. Estimation of regional-scale groundwater flow properties in the Bengal Basin of India and Bangladesh. *Hydrogeol. J.* **17**, 1329–1346 (2009).

[47] Michael, H. A. & Voss, C. I. Controls on groundwater flow in the Bengal Basin of India and Bangladesh: regional modeling analysis. *Hydrogeol. J.* **17**, 1561–1577 (2009).

[48] Phan, H. V., Van Geer, F. C., Bui Tran, V., Dubelaar, W. & Oude Essink, G. H. P. Paleo-hydrogeological reconstruction of the fresh-saline groundwater distribution in the Vietnamese Mekong Delta since the late Pleistocene. *J. Hydrol. Reg. Stud.* **23**, 100594 (2019).

[49] Jurgens, B. C. et al. Over a third of groundwater in USA public-supply aquifers is Anthropocene-age and susceptible to surface contamination. *Commun. Earth Environ.* **3**, 153 (2022).

[50] Wang, R., Colombera, L. & Mountney, N. P. Geological controls on the geometry of incised-valley fills: insights from a global dataset of late-Quaternary examples. *Sedimentology* **66**, 2134–2168 (2019).

[51] Stanley, D. J. & Warne, A. G. Worldwide initiation of holocene marine deltas by deceleration of sea-level rise. *Science* **265**, 228–231 (1994). 17750665 10.1126/science.265.5169.228

[52] Rodell, M. & Reager, J. T. Water cycle science enabled by the GRACE and GRACE-FO satellite missions. *Nat. Water* **1**, 47–59 (2023).

[53] Korus, J. T., Joeckel, R. M., Divine, D. P. & Abraham, J. D. Three-dimensional architecture and hydrostratigraphy of cross-cutting buried valleys using airborne electromagnetics, glaciated Central Lowlands, Nebraska, USA. *Sedimentology* **64**, 553–581 (2017).

[54] Knight, R. et al. Airborne geophysical method images fast paths for managed recharge of California’s groundwater. *Environ. Res. Lett.* **17**, 124021 (2022).

[55] Jørgensen, F. et al. Transboundary geophysical mapping of geological elements and salinity distribution critical for the assessment of future sea water intrusion in response to sea level rise. *Hydrol. Earth Syst. Sci.* **16**, 1845–1862 (2012).

[56] Fitterman, D. V. & Deszcz-Pan, M. Helicopter EM mapping of saltwater intrusion in Everglades National Park, Florida. *Explor. Geophys.* **29**, 240–243 (1998).

[57] Ball, L. B., Davis, T. A., Minsley, B. J., Gillespie, J. M. & Landon, M. K. Probabilistic categorical groundwater salinity mapping from airborne electromagnetic data adjacent to California’s Lost Hills and Belridge oil fields. *Water Resour. Res.* **56**, e2019WR026273 (2020).

[58] Kirkegaard, C., Sonnenborg, T. O., Auken, E. & Jørgensen, F. Salinity distribution in heterogeneous coastal aquifers mapped by airborne electromagnetics. *Vadose Zone J.* **10**, 125–135 (2011).

[59] Burton, B. L. et al. Airborne electromagnetic, magnetic, and radiometric surveys of the Mississippi Alluvial Plain and Chicot Aquifer System, March 2018 and May - August 2021. *U.S. Geological Survey data release*. 10.5066/P9KPK3UJ (2024).

[60] Brodie, R. ga-aem: modelling and inversion of airborne electromagnetic (AEM) data in 1D. Github. https://github.com/GeoscienceAustralia/ga-aem/releases/tag/Release-20160606 (2024).

[61] Oldenburg, D. W. & Li, Y. Estimating depth of investigation in DC resistivity and IP surveys. *Geophysics* **64**, 403–416 (1999).

[62] Christiansen, A. V. & Auken, E. A global measure for depth of investigation. *Geophysics* **77**, WB171–WB177 (2012).

[63] Clark, B. R. et al. National-scale grid to support regional groundwater availability studies and a national hydrogeologic database. *U.S. Geological Survey data release*. 10.5066/F7P84B24 (2018).

[64] Deutsch, C. V. & Journel, A. G. *GSLIB: Geostatistical Software Library and User’s Guide* (Oxford University Press, 1997).

[65] Waelbroeck, C. et al. Sea-level and deep water temperature changes derived from benthic foraminifera isotopic records. *Quat. Sci. Rev.* **21**, 295–305 (2002).

