# Supplementary material for: The saline groundwater legacy of a large buried coastal paleo-estuary
Source: Nat Commun. 2026 Jul 27;17:9119. doi: 10.1038/s41467-026-76005-5 (PMC13518967; doi:10.1038/s41467-026-76005-5)
Supplement: Supplementary file 1 [file 41467_2026_76005_MOESM1_ESM.pdf]

Supplementary Information

The saline groundwater legacy of a large buried coastal paleo-estuary

Burke J. Minsley[1*], Holly A. Michael[2], Max A. Lindaman[3], Lyndsay B. Ball[1], Stephanie R. James[1], JR Rigby[4], Wade H. Kress[5], Frank T.-C. Tsai[6], Bennett E. Hoogenboom[1†]

[1]U.S. Geological Survey, Geology, Geophysics, and Geochemistry Science Center, Denver, CO

[2]University of Delaware, Department of Earth Sciences, Department of Civil, Construction, and Environmental Engineering, Newark, DE

[3]U.S. Geological Survey, Lower Mississippi-Gulf Water Science Center, Baton Rouge, LA

[4]U.S. Geological Survey, Lower Mississippi-Gulf Water Science Center, Oxford, MS

[5]U.S. Geological Survey, Lower Mississippi-Gulf Water Science Center, Nashville, TN

[6]Louisiana State University, Department of Civil and Environmental Engineering, Baton Rouge, LA

[†]present address: U.S. Geological Survey; California Water Science Center; Sacramento, CA 95819, USA.

*Corresponding author: Burke Minsley, bminsley@usgs.gov

Contents:

Supplementary Discussion

Supplementary Table S1

Supplementary Figures 1-4

Supplementary References

Supplementary Discussion

The combined influence of salinity and lithology on resistivity

Bulk electrical resistivity can be controlled by both pore fluid salinity and lithology, complicating interpretations when both underlying properties vary. While Archie's Law[1] accounts for effects of the saturating pore fluid including porosity, saturation, and fluid conductivity, this commonly used model does not include effects of surface conduction that can strongly influence bulk resistivity especially in the presence of clays. Models that include both pore-fluid and surface conduction effects[2] more accurately reflect the covarying response of resistivity to both parameters.

Theoretical curves showing bulk resistivity as a function of both fluid conductivity and surface conductivity are shown as dashed curves in Supplementary Fig. 1 using a porosity of 0.3 and cementation exponent of 1.5 representative of unconsolidated sediments. Increasing surface conductivity, a proxy for increased clay fraction, can decrease bulk resistivity by several orders of magnitude compared with Archie's Law ($\sigma_s = 0$) at low fluid conductivity that is representative of fresh groundwater systems. At high salinity, however, the influence of surface conductivity becomes minimal compared to that of fluid conductivity as changes in bulk resistivity are primarily driven by the pore fluid. While fluid resistivity is also sensitive to temperature, there is only about a 2% decrease in resistivity for every one degree increase in temperature[3]. A decrease in temperature of 10 degrees Celsius for a material with original bulk resistivity of 50 ohm-m would result in a resistivity of 60 ohm-m, which is minimal compared with the variability observed as a function of varying fluid salinity or clay fraction and is therefore not expected to be a significant factor in the results of this study.

For a given fluid salinity, bulk resistivity decreases with increasing porosity and/or increasing surface conductivity due to increasing clay fraction. Points within the shaded region of Supplementary Fig. 1 indicate a bulk resistivity that is lower than the fluid resistivity, requiring the influence of surface conduction. Points above the shaded region may be controlled in part by surface conduction, reducing the bulk resistivity, but also could be explained by higher porosity without surface conduction or by the discrepancy in sampling volumes of the fluid conductivity at boreholes versus the larger 1 km-scale bulk resistivity from AEM data.

Supplementary Tables

Supplementary Table 1. Summary of approximate resistivity ranges for different combinations of fluid salinity and clay content based on the curves in Supplementary Fig. 1. SC = specific conductance. σ_s = surface conductivity.

	**Low salinity (SC < 1000 µS/cm)**	**Moderate salinity (SC 1000-10000 µS/cm)**	**High salinity (SC >10000 µS/cm**
**Low clay (σ_s < 0.001 S/m)**	50 – 1000+ ohm-m	4 – 100 ohm-m	<1 – 10 ohm-m
**Moderate clay (σ_s ~0.01 S/m)**	30-200 ohm-m	4 – 80 ohm-m	<1 – 10 ohm-m
**High clay (σ_s ~ 0.1 S/m)**	5-30 ohm-m	2 – 20 ohm-m	<1 – 8 ohm-m

Supplementary Figures

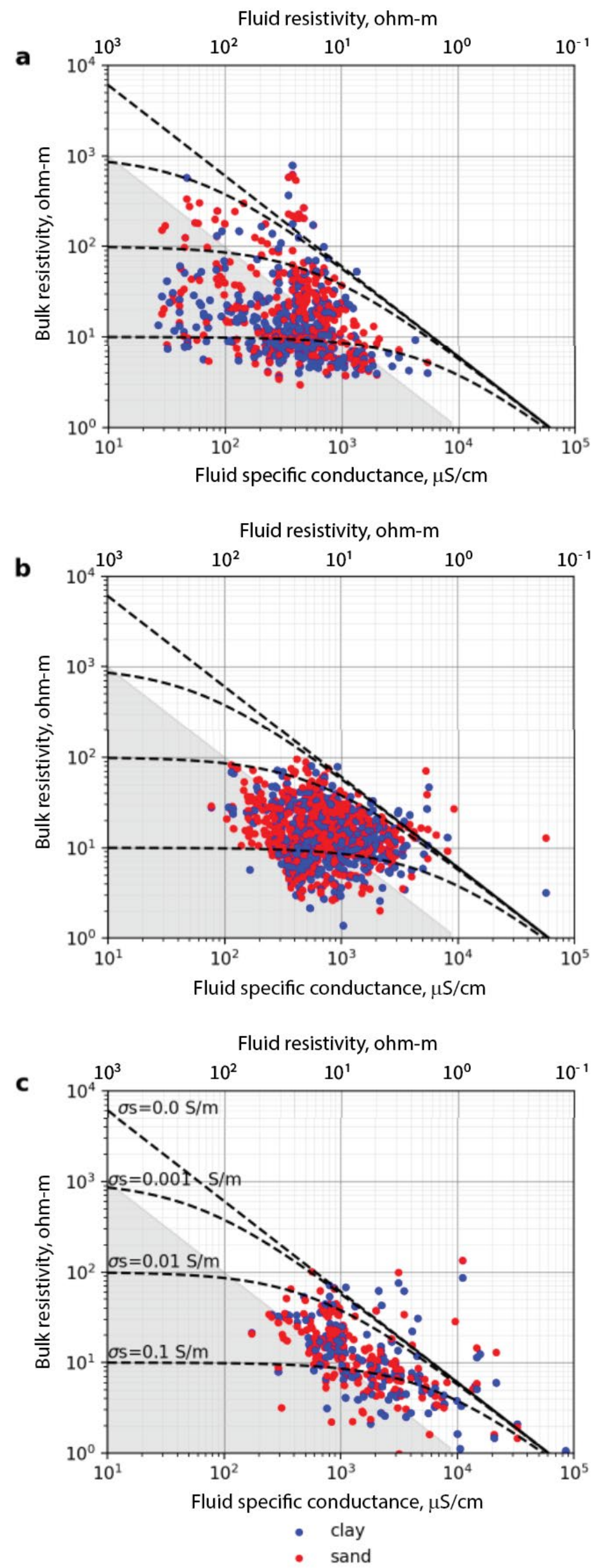

Supplementary Fig. 1. Relationship between borehole specific conductance (SC) and AEM-derived electrical resistivity in the lower Mississippi River Valley. Results are separated into three latitude ranges: a) 29.35766-30.07888°, b) 30.07888-30.48406°, and c) 30.48406-30.97880°. Points are colored by the dominant lithology predicted at their respective locations by a three-dimensional lithologic model[4], with blue representing clay-dominated and red is sand-dominated. Theoretical curves that include different levels of surface conductivity (σ_s) are shown with dashed black lines, with Archie's Law representing the diagonal with $\sigma_s = 0$. Light grey shading indicates the region where AEM resistivity is equal to or less than specific conductance, requiring surface conduction contributions to the bulk resistivity.

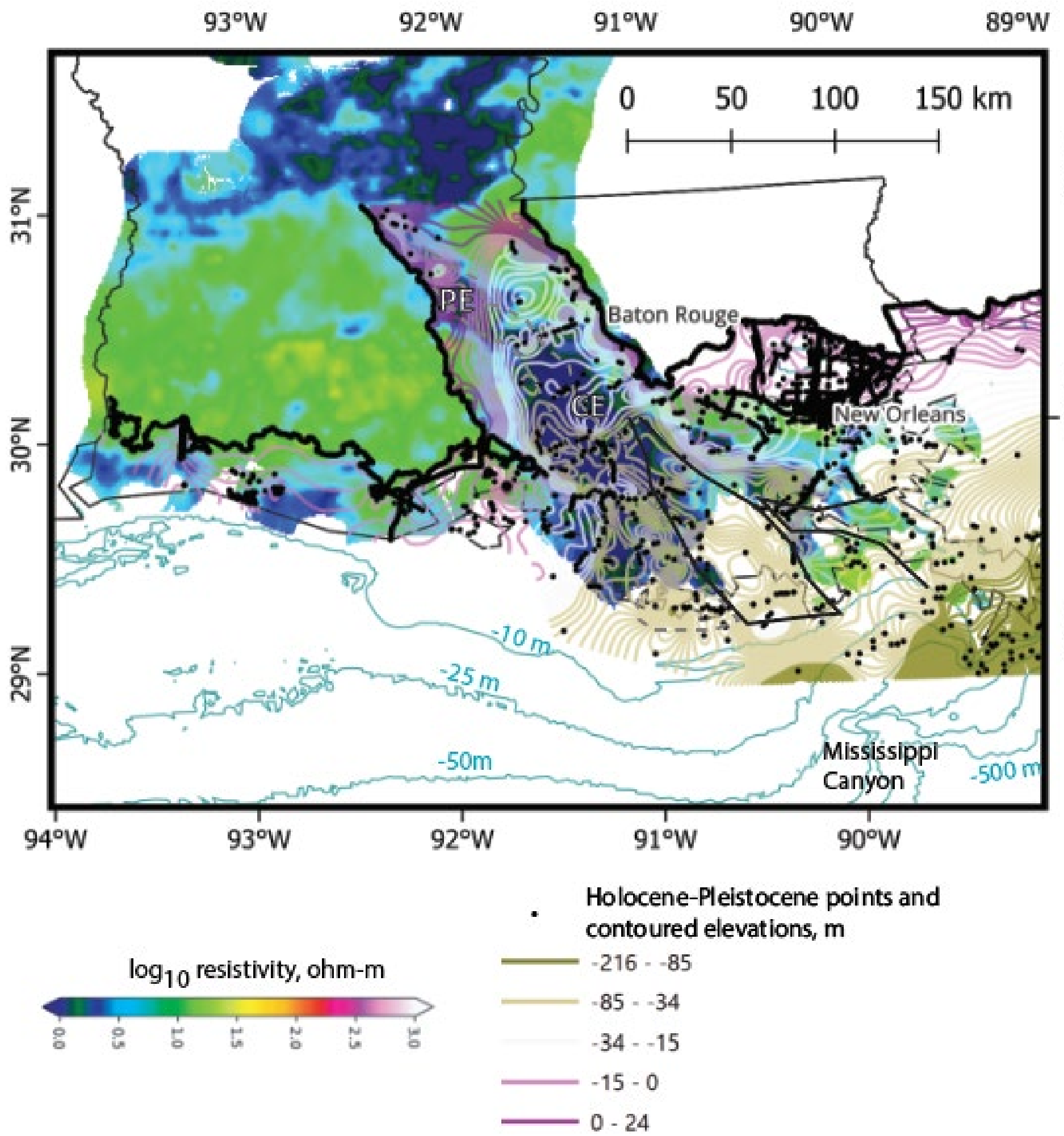

Supplementary Fig. 2. Comparison of AEM and Holocene-Pleistocene surface. Resistivity models at 170 – 175 m below sea level are shown together with the points and associated contours indicating the elevation of the Holocene-Pleistocene boundary[5].

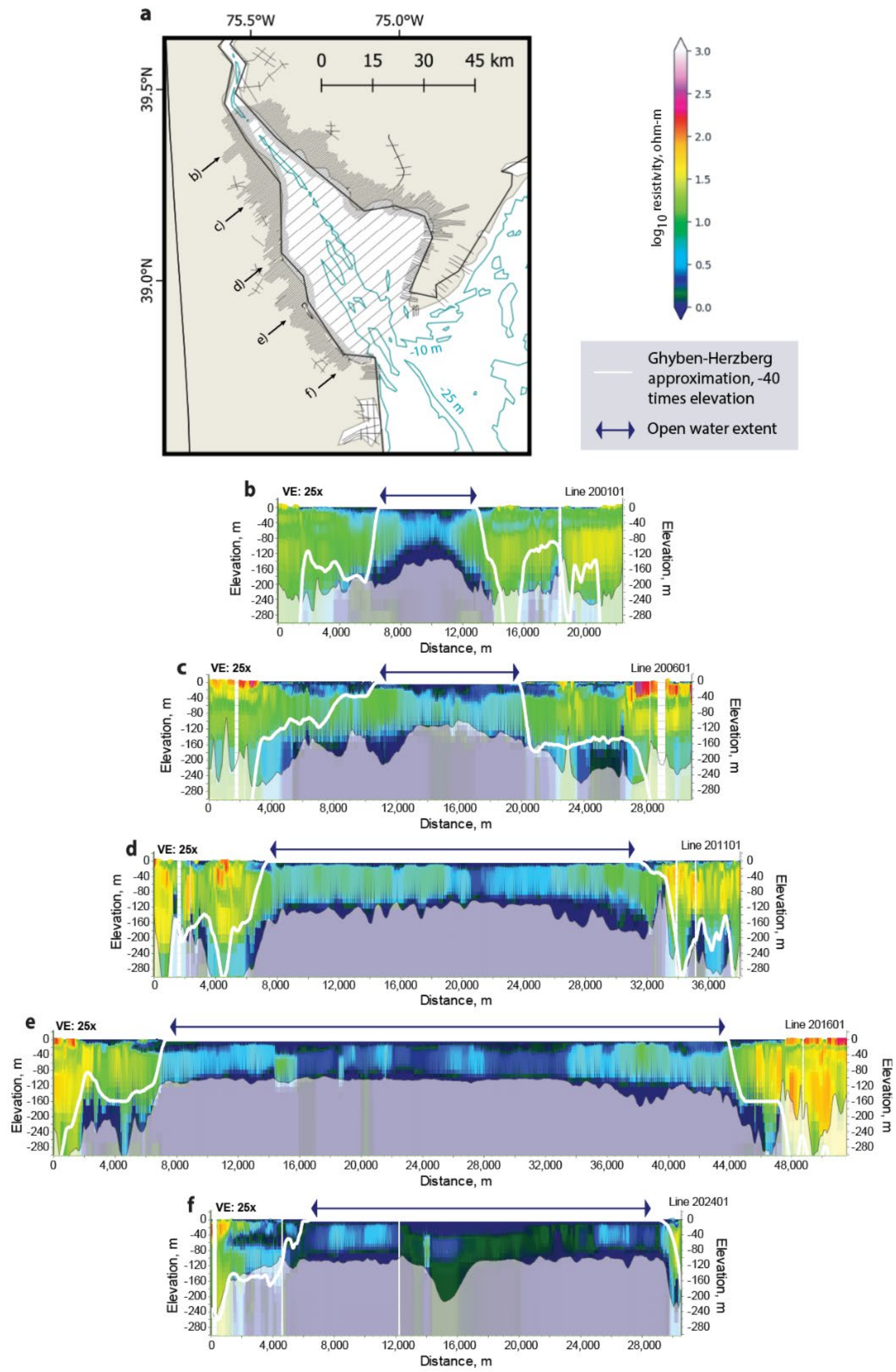

Supplementary Fig. 3. Additional resistivity cross-sections from Delaware Bay. Resistivity profiles show correspondence with deep resistivity patterns and the predicted location of the theoretical Ghyben-Herzberg interface. White transparency shading towards the bottom of each profile indicates the depth of investigation where resistivity values are not well-constrained by data. VE = vertical exaggeration.

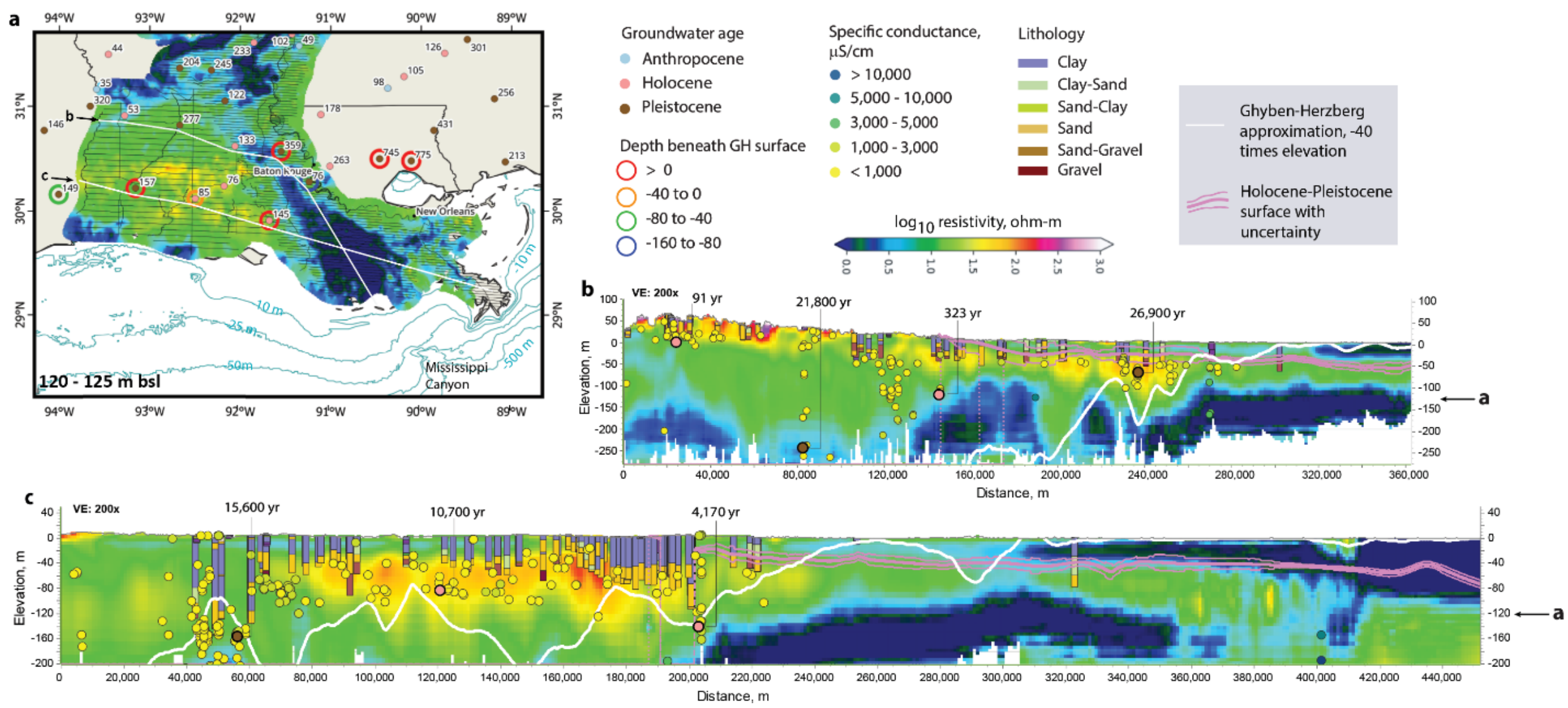

Supplementary Fig. 4. Comparison with groundwater age data. a) Resistivity depth slice at 120 – 125 m bsl with groundwater age measurement locations[6]. Points are colored by age[6], with a surrounding circle that indicates the measurement depth (labeled as numbers next to each point) relative to the theoretical GH depth at that location. b-c) Resistivity cross-sections along irregular profiles show the vertical position of groundwater age measurements[6] relative to the GH surface. Regions of low resistivity above the GH line, or elevated resistivity beneath the GH line, indicate areas where the distribution of groundwater salinity is out of equilibrium. Age measurements in c) are just below or just above the GH line, and correspond with areas of low groundwater salinity measurements, suggesting that wells are tapping relic fresh water. VE = vertical exaggeration.

Supplementary References

1. Archie, G. E. The electrical resistivity log as an aid in determining some reservoir characteristics. *Trans. Amer. Inst. Mining Metallurgical and Petroleum Engineers* **146**, 54–62 (1942).
2. Waxman, M. H. & Smits, L. J. M. Electrical conductivities in oil-bearing shaly sands. *Society of Petroleum Engineers Journal* **8**, 107–122 (1968).
3. Sen, P. N. & Goode, P. A. Influence of temperature on electrical conductivity on shaly sands. *Geophysics* **57**, 89–96 (1992).
4. Yang, S., Tsai, F. T.-C. & Yarbrough, L. D. Horizon-assisted lithologic modeling: understanding Mississippi Embayment and Coastal Lowlands aquifer systems in Louisiana and southwestern Mississippi, USA. *Hydrogeology Journal* **32**, 1447–1470 (2024).
5. Heinrich, P., Paulsell, R., Milner, R., Snead, J. & Peele, H. Investigation and GIS development of the buried Holocene-Pleistocene surface in the Louisiana coastal plain. *Louisiana Geological Survey-Louisiana State University for Coastal Protection and Restoration Authority of Louisiana* (2015).
6. Jurgens, B. C. *et al.* Over a third of groundwater in USA public-supply aquifers is Anthropocene-age and susceptible to surface contamination. *Communications Earth & Environment* **3**, 153 (2022).
