# Supplementary material for: The saline groundwater legacy of a large buried coastal paleo-estuary
Source: Nat Commun. 2026 Jul 27;17:9119. doi: 10.1038/s41467-026-76005-5 (PMC13518967; doi:10.1038/s41467-026-76005-5)
Supplement: Supplementary file 2 [file 41467_2026_76005_MOESM2_ESM.pdf]

nature portfolio

Peer Review File

The saline groundwater legacy of a large buried coastal paleo-estuary

Corresponding Author: Dr Burke Minsley

This file contains all reviewer reports in order by version, followed by all author rebuttals in order by version.

Version 0:

Reviewer comments:

Reviewer #1

(Remarks to the Author)
This manuscript presents a compelling synthesis of large-scale airborne electromagnetic (AEM) surveys in coastal Louisiana and Delaware Bay to map subsurface salinity distributions and interpret their geological origins. The authors identify a large (>10,000 km²) region of elevated groundwater salinity beneath coastal Louisiana's deltaic plain that they attribute to residual seawater emplaced during post-glacial drowning of an incised Mississippi River valley. A novel space-for-time comparison with modern Delaware Bay, itself a drowned river valley estuary, provides supporting context for their interpretation. The datasets are vast in scale, the geological narrative is well-constructed, and the implications for coastal groundwater management are significant. I recommend acceptance with minor revisions. The study represents an important contribution to understanding legacy salinity in coastal aquifer systems and demonstrates the power of regional-scale AEM for hydrogeological characterization. I offer minor commnents below that may help strengthen the manuscript.
Comments
• The Extended Data Fig. 1 analysis of resistivity versus specific conductance is informative. It may be worth noting explicitly in the main text that the surface conductivity contribution from clays is small relative to pore-fluid conductivity in the brackish-to-saline regime, which strengthens confidence that the low-resistivity features central to this study are probably salinity-dominated. The clay-versus-salinity ambiguity is primarily relevant in the fresh groundwater zones, where it could affect lithologic interpretation but does not impact the main conclusions.
• The literature review notes that "past studies have identified the presence of Holocene seawater in buried paleovalleys, supported by borehole data, modeling, geochemical observations, and geophysical surveys" (refs 11–14). A recent study by Le et al. (2025, Nature Communications) used deep-sensing magnetotelluric soundings to image saline groundwater within the incised Ganges paleovalley in coastal Bangladesh, flanked by preserved Pleistocene freshwater bodies; this is a setting with clear parallels to the paleo-estuary and paleo-fresh groundwater features described here. This work could be cited alongside refs 11–14.
• The paper states this is "the first study where a unique space-for-time approach informed on multiple AEM studies links present and past subsurface properties and processes." This is a strong claim. The space-for-time substitution is creative and well-executed, but the authors should acknowledge its limitations; the two systems differ substantially in scale, sediment supply, tectonic setting, and climate history. The comparison is illustrative rather than strictly quantitative.
• The methods describe a weighted merging of the fixed-wing Tempest (time-domain, 120 m flight height) and helicopter Resolve (frequency-domain, 30–40 m flight height) datasets using a depth-dependent weighting function that transitions from ~50/50 at the surface to 100% Tempest by 75 m. Was there any formal comparison at coincident or nearby locations to validate the consistency of the two datasets and the choice of merging weights?
• The authors note that the buried salinity is "out of equilibrium" with modern hydrologic conditions and that flushing would be expected over time, but no estimate of flushing timescales is provided. Even an order-of-magnitude estimate or citation to relevant modeling work would help the reader assess how persistent this legacy salinity is and over what timeframe it might impact water resource management.
• The paper demonstrates a compelling framework linking AEM imaging with space-for-time comparison to interpret paleo-salinity. A brief discussion of whether this approach could be applied predictively to other major delta systems globally (e.g., Mekong, Nile, Irrawaddy) where similar post-glacial histories may have left analogous buried salinity features would strengthen the paper's broader impact, particularly for the Nature Water readership.
• In the Methods, the DOI estimation for the Tempest data follows Oldenburg and Li (1999), while the Aarhus Workbench inversions presumably use a different DOI metric. Are these comparable? Some comment on the consistency of DOI estimates across the different systems and inversion approaches would be helpful.

• Minor text issues: "~10,00 km²" appears to be a typo for "~10,000 km²" in the Delaware Bay comparison section.

Reviewer #2

(Remarks to the Author)
The authors have collected very impressive AEM datasets from Mississippi Delta and Delaware Bay. Their data clearly show a 3D image of high and low resistivity strata corresponding to saline and fresh groundwater in the Mississippi Delta (MD). They also provide a similar 3D image of resistivity in Delaware Bay to support their interpretation. They produce a very convincing model for the formation of deep saline water in the PE and CE along the Mississippi incised valley, followed by fresh water displacing the shallower saline water. I have not seen such extensive displacement of saline water documented like this. This paper is noteworthy and of broad interest, and should clearly be published. However, we have some suggests for improving the manuscript and increasing its readability that is needed before publication. The readability could be improved, perhaps because the presentation of the data and its interpretation was mingled, I needed multiple read throughs to fully understand the paper, although it was worth the effort,

An addition that would really help for understanding the interpretation of the inversion in the MD would be a contour map of the actual incised valley, e.g., depth to base of Holocene. It would show where the incised valley is and how deeply it cut through the preexisting strata. You should be able to construct it from the Saucier and Wessels references along with other publications such as Heinrich et al., 2015 and Jankowski et al., 2017. The depth of the Holocene/Pleistocene boundary can also be added to the sections in Figure 2 e, f, g.

In Figure 2 add scale to the right side of the transects as well to make it easier to see the depths of the features. The map being in degrees and the section in km, also makes it harder to correlate them. I suggest adding some longitude ticks to the X axis of the sections.

I agree that groundwater salinity is the primary control on conductivity, but the Extended Data Fig.1 shows a significant clay effect. A large portion of points stay within the shaded area. Therefore, I suggest specifying the resistivity range for fresh, brackish, and saline water to clarify clay effects on groundwater conductivity, perhaps as a table.

On the right side of section g, there is a deeply incised saline wedge extending from the surface to over 100 m that is not discussed. I got confused as I initially thought of this as the incised valley. It is highlighted in Figure 5. Is it a saline wedge from the abandonment of the St. Bernard lobe? Please include this in the presentation and discuss its origin.

An important part of the model is the substantial flow of PFW above the PE and CE. The areas appear to be sandy (Ex. Data Fig. 3). At the same time, you say "The present-day hydrologic conditions would tend to flush the saline water out of the system, but the presence of low-permeability materials and the slow rate of groundwater flow within deep aquifer systems allow the saltwater to persist". You have shallower parts flushed in your interpretation and deeper not. The recent Voller et al. (2025) modeling paper shows that an aquitard may be needed to produce this type of flow boundary. Can you include rough calculations about the permeability of sandy aquifers and muddy aquitards that show where the flow of fresh water could or could not displace 10s of km of saline water during the Holocene? This flow is an important part of the model shown in Figure 4. Is there any evidence of flushing such as boron isotopes in wells (Ravenscroft et al., 2004)?

Additional comments: [text is brackets are suggested additions]
Line 44 - freshwater-saltwater interface[, in the absence of other factors,] is determined by the modern equilibrium … Also add a reference.
Line 50 - led to the formation of deeply incised river valleys – provide a little more description than just "led".
Line 52 - incised valleys are filled with fluvially sediments as well as glacially derived ones.
Line 53 - These infilled valleys can be [fine-grained barriers or] permeable pathways
Line 119 - Why is 10 ohm-m chosen as the lower bound for the aquifer? Can you associate it with a particular salinity?
Line 131-2. "… which we interpret as an older paleo-estuary (PE)". This was confusing as the PE was formed in the same transgression as is made clear a few sentences later. Perhaps: which we interpret [as a] paleo-estuary (PE) [formed by flooding of the incised valley].
Line 133 – How steeply dipping in the Pleistocene? They may be more steeply dipping than the coastal plain, but are probably still a pretty low dip. Either correct or provide dips.
Line 134 – 'fall line'. I don't know the usage on the Gulf Coast. Along the East Coast the fall line is at the edge of the Mesozoic coastal plain. West of the Mississippi, the appear to be two topographic breaks, one at the edge of the Mesozoic and one closer to the shore that is identified as the fall line. I don't know which one is properly called the fall line.
Line 157 – for the moderately low-resistivity halo, perhaps a different set of initials to distinguish it from the deeper PE (e.g., PEh = PE halo or PPE = perched PE).
Line 205 – Again, the dips are not steep, although perhaps steep for GW surfaces. All the sections for MD and DB should have the same Vertical Exaggeration, which should be labeled. Fig. 3d is clearly different from e and f. The two locations are of sufficiently different scale that they cannot be easily shown with the same V.E., which is why each figure should be labeled.
Line 208 – In cross section view (Fig. 3c, d-f -> 3c is not a cross section.
Line 236 – perhaps a river valley parallel section could be added to Figure 2 or 5 to correspond to the one in Figure 4.
Line 248 – Which resistivity value used here to define the top of low-resistivity feature?
Line 248 – Why do you describe it as "multiple stages of valley filling"? I see one progressive valley filling and a flow of fresh water displacing the shallower saline water.
Line 258 – "of poor water quality" probably better described as non-potable saline water.

Line 278 - Another recent imaging of a paleovalley by EM is Le et al., 2025.
Figure 2 - The unit of specific conductance can be converted to resistivity (ohm.m) for direct comparison to resistivity values from AEM, even though they measure different properties (bulk resistivity versus pore fluid). Add IV and TS in f and PE in g. Plot all sections (e, f, g) at the same scale
Figure 3. Typo in 'Open Water Extent" in legend. Again use the same scales in the 3 sections. The confining layers likely bend upwards under the bay, as the sediment loading would depress them on the flanks. It is likely that some confining layers correspond to the boundary between the PFW inflow and the high resistivity below.
Figure 4. Typo in a: perpendicular. Present-day coastline in a should align with section g and be at top of section. Add PWF for areas where fresh water has displaced salt water. Are the dashed dark blue lines schematic mixing flow in salt water wedge? This should be indicated in the legend.
Figures 1, 2, 3, 5 label some of the water depth contours.
Extended Data Figure 1. Why is a significant amount of clay-dominated lithology not within the shaded area, where surface conduction of clay is required to explain conductivity of sediments not obeying Archie's law? Using the same unit: ohm.m or S/m would be better for this demonstration. Or could have two x-axis labels. Which is Archie's law equation or version that you use to infer this? Need references and specifying parameters used in the equation, such as temperature. You might consider adding a table to classify specific groups: freshwater- vs saltwater-regions, clay outside the shaded region versus sand inside the shaded region. I found this graph is useful to interpret freshwater/saltwater distribution and clay effects. However, you should mention its limitations such as not considering temperature and the mismatch between the resolution scale between AEM and SC.
Figure 5. You could add the GW age values above sections b, c to help compare the map and section view.

References

Heinrich, P., Paulsell, R., Milner, R., Snead, J. & Peele, H. Investigation and GIS development of the buried Holocene-Pleistocene surface in the Louisiana coastal plain. p.140 Louisiana Geological Survey and Louisiana State University for the Louisiana Coastal Protection and Restoration Authority (2015).

Jankowski, K. L. et al. (2017) Vulnerability of Louisiana's coastal wetlands to present-day rates of relative sea-level rise. Nat. Commun. 8, 14792 doi: 10.1038/ncomms14792.

Le, H.D., K. Key, M.S. Steckler, N. Sazeed, M. Person, A. Bhuiyan, M.R. Khan and K.M. Ahmed (2025). Buried deep freshwater reserves beneath salinity-stressed coastal Bangladesh, Nature Comm., 16, 10740, https://doi.org/10.1038/s41467-025-65770-4.

Ravenscroft P. and J.M McArthur, 2004, Mechanism of regional enrichment of groundwater by boron: the examples of Bangladesh and Michigan, USA, Applied Geochemistry, 19, 1413-1430, https://doi.org/10.1016/j.apgeochem.2003.10.014.

Voller, V. R., Sazeed, N., Luong, L., Camille, A., Steckler, M., Leary, K., & Person, M. (2025). Onshore entrapment of seawater in coastal aquifers by rapid coastline progradation. Water Resources Research, 61, e2024WR038157. https://doi.org/10.1029/2024WR038157

Reviewer #3

(Remarks to the Author)
I co-reviewed this manuscript with one of the reviewers who provided the listed reports. This is part of the Nature Communications initiative to facilitate training in peer review and to provide appropriate recognition for Early Career Researchers who co-review manuscripts.

Reviewer #4

(Remarks to the Author)
Dear editor and authors,
The paper describes paleo-saline groundwater volumes for two (large) study areas (large buried coastal paleo-estuaries) along the coast of the USA, based on large-scale airborne electromagnetic measurements (AEM). I've read the paper with pleasure but the findings are very site specific and the findings for general scientific interest are not novel.
The main findings as summarized in the abstract are (literally cited):

- Finding 1: "Coastal groundwater management typically focuses on preventing intrusion from modern sources of seawater; however, past geological processes can also leave a legacy of saline groundwater now hidden in the subsurface.".
This is common sense and well known for many delta's in the world like the Dutch delta and the Nile delta. Aquifers of these delta's contain paleo-saline groundwater infiltrated during their geological development and transgressions in the Holocene and earlier inter-glacial periods. Nowadays, they face major challenges in groundwater management for freshwater availability for agriculture and drinking water.
- Finding 2: "Here we show, for the first time using multiple extensive airborne electromagnetic (AEM) surveys, detailed evidence of the residual salinity from a paleo-estuary filling a late-Pleistocene incised valley impacting more than 10,000 km2 that is now hidden beneath coastal Louisiana's deltaic plain."
This is very site specific, though a nice example, besides many others. Airborne electromagnetic (AEM) surveys for large scale mapping the salinity of the subsurface have been carried out since the beginning of this century and is a well known and proven method published in many papers.

- Finding 3: “Our results show that the three-dimensional pattern of saline groundwater beneath Louisiana mimics that of near-surface aquifers surrounding the modern Delaware Bay estuary, fingerprinting the signature of the past drowning of a large, incised valley of the Mississippi River following post-glacial sea-level rise.
This finding is very site specific, see comments for finding 2.

- Finding 4: “These findings demonstrate a new framework for understanding legacy sources of saltwater critical for managing stressed water resources along global coastlines.”
There is no new framework demonstrated. They demonstrated for 2 study areas the use of AEM in combination with ground-truth data to map and understand the paleo-saline groundwater occurrences.

To summarize, the findings presented in the paper are site specific and the general ideas and applied methodology cannot be judged as novel. It is common sense that paleo-saline groundwater occurrences below deltas resulted from past transgressions and occur widespread globally being critical for freshwater availability. Using large scale AEM for mapping those paleo saline groundwater bodies is regularly done since the past 15 years. Therefore, I will not recommend publication in Nature.

Version 1:

Reviewer comments:

Reviewer #2

(Remarks to the Author)
The authors edited to text to respond to our comments, and those of the other reviewers. The manuscript addressed our issues and is now improved and easier to read. I just had a few additional minor suggested changes and now recommend the article for publication.

Change:
This low-resistivity feature continues to narrow inland until terminating in a narrow channel emerging from more steeply dipping low-resistivity Pleistocene and older units (~4–13 m/km, compared with just ~0.2 m/km for the H-P surface) that mark an interpreted ‘fall line’ (FL) marking the transition from upland alluvial to coastal plain regions (d).
To:
This low-resistivity feature continues to narrow inland until terminating at the interpreted ‘fall line’ (FL) which marks the upland alluvial to coastal plain transition (UCT; dashed line). This coincides with a change from low-resistivity Pleistocene units farther north dipping at (~4–13 m/km, compared with just ~0.2 m/km for the H-P surface.
Since the FL and UCT are on the map, but the dips are not,identify with those first, then add the dip comments.

Change:
This inferred paleo-estuary may have formed during the early stages of marine transgression that first filled deeply entrenched channels in this region33 before continued sea-level rise filled the coastal embayment (CE) that occupies a much larger area of nearly 10,000 km2.
To:
This inferred paleo-estuary may have filled during the early stages of marine transgression33 before continued sedimentation during sea-level rise filled the coastal embayment (CE) that occupies a much larger area of nearly 10,000 km2.
It is the sediments, not the SLR that fill the estuary

Better to call TS topstratum and then describe it as a clay cap, that say clay cap (TS).

Figure 2. Label of profile h is missing. Explanation of PCL and UCT are missing from caption.

Reviewer #3

(Remarks to the Author)
I co-reviewed this manuscript with one of the reviewers who provided the listed reports. This is part of the Nature Communications initiative to facilitate training in peer review and to provide appropriate recognition for Early Career Researchers who co-review manuscripts.

Reviewer #4

(Remarks to the Author)
Based on the authors’ response, the positive assessments provided by the other reviewers, and the overall quality of the manuscript, I no longer consider my earlier concern regarding the site-specific nature of the study to constitute a valid argument against publication.
The extensive spatial scale of the AEM survey, which facilitates the derivation of system-scale insights—particularly with respect to the development of one of North America’s largest incised valley systems—renders the study both scientifically robust and broadly relevant. I agree with the authors’ response that it is necessary to describe results from specific places in

order to make unique observations, which are essential for developing new insights.
In light of these considerations, I support the publication of this manuscript in Nature Communications.

Open Access This Peer Review File is licensed under a Creative Commons Attribution 4.0 International License, which permits use, sharing, adaptation, distribution and reproduction in any medium or format, as long as you give appropriate credit to the original author(s) and the source, provide a link to the Creative Commons license, and indicate if changes were made.
In cases where reviewers are anonymous, credit should be given to 'Anonymous Referee' and the source.
The images or other third party material in this Peer Review File are included in the article's Creative Commons license, unless indicated otherwise in a credit line to the material. If material is not included in the article's Creative Commons license and your intended use is not permitted by statutory regulation or exceeds the permitted use, you will need to obtain permission directly from the copyright holder.
To view a copy of this license, visit https://creativecommons.org/licenses/by/4.0/

Review responses below in blue
Burke Minsley
22 May 2026

REVIEWER COMMENTS

Reviewer #1 (Remarks to the Author):

This manuscript presents a compelling synthesis of large-scale airborne electromagnetic (AEM) surveys in coastal Louisiana and Delaware Bay to map subsurface salinity distributions and interpret their geological origins. The authors identify a large (>10,000 km²) region of elevated groundwater salinity beneath coastal Louisiana's deltaic plain that they attribute to residual seawater emplaced during post-glacial drowning of an incised Mississippi River valley. A novel space-for-time comparison with modern Delaware Bay, itself a drowned river valley estuary, provides supporting context for their interpretation. The datasets are vast in scale, the geological narrative is well-constructed, and the implications for coastal groundwater management are significant. I recommend acceptance with minor revisions. The study represents an important contribution to understanding legacy salinity in coastal aquifer systems and demonstrates the power of regional-scale AEM for hydrogeological characterization. I offer minor commnents below that may help strengthen the manuscript.

Thank you for the positive review

Comments

• The Extended Data Fig. 1 analysis of resistivity versus specific conductance is informative. It may be worth noting explicitly in the main text that the surface conductivity contribution from clays is small relative to pore-fluid conductivity in the brackish-to-saline regime, which strengthens confidence that the low-resistivity features central to this study are probably salinity-dominated. The clay-versus-salinity ambiguity is primarily relevant in the fresh groundwater zones, where it could affect lithologic interpretation but does not impact the main conclusions.

Added clarifying sentence along these lines after introducing Extended Data Fig. 1 (now Supplementary Fig. 1): “The ambiguity in resistivity due to clay versus salinity is primarily relevant in fresh groundwater zones; at higher salinity the effect of clay on resistivity is reduced.”

• The literature review notes that "past studies have identified the presence of Holocene seawater in buried paleovalleys, supported by borehole data, modeling, geochemical observations, and geophysical surveys" (refs 11–14). A recent study by Le et al. (2025, Nature Communications) used deep-sensing magnetotelluric soundings to image saline groundwater within the incised Ganges paleovalley in coastal Bangladesh, flanked by preserved Pleistocene freshwater bodies; this is a setting with clear parallels to the paleo-estuary and paleo-fresh groundwater features described here. This work could be cited alongside refs 11–14.

Absolutely—this paper came out around the time of our initial submission and therefore wasn't cited. But it certainly has important similarities to our study and is added to the citations where you suggest. Related to the previous comment, we also note that Le et al. state that "We neglected the effects of clay content on formation resistivity since saltwater conductivity is the dominant factor." We believe the additional discussion of clay and pore fluid impacts included in the supplementary information adds important interpretational context in our study that is often overlooked in the literature.

• The paper states this is "the first study where a unique space-for-time approach informed on multiple AEM studies links present and past subsurface properties and processes." This is a strong claim. The space-for-time substitution is creative and well-executed, but the authors should acknowledge its limitations; the two systems differ substantially in scale, sediment supply, tectonic setting, and climate history. The comparison is illustrative rather than strictly quantitative.

Modified 1st sentence in last paragraph of section describing Delaware Bay as suggested:

"The spatial patterns of salinity interpreted from AEM-derived resistivity data surrounding Delaware Bay share many similarities with observations in coastal Louisiana, allowing for an illustrative comparison despite differences in scale, sediment supply, tectonic setting, and climate history that may also be relevant in other coastal Holocene incised valleys globally"

• The methods describe a weighted merging of the fixed-wing Tempest (time-domain, 120 m flight height) and helicopter Resolve (frequency-domain, 30–40 m flight height) datasets using a depth-dependent weighting function that transitions from ~50/50 at the surface to 100% Tempest by 75 m. Was there any formal comparison at coincident or nearby locations to validate the consistency of the two datasets and the choice of merging weights?

We compared the merged Resolve and Tempest results with existing boreholes and regional hydrostratigraphic surfaces to ensure visual consistency at the 1km x 1km gridding scale, though the specific weight values were not calibrated or optimized. Instead, we sought a roughly equal blend of the two systems with the depth of investigation of the Resolve system (upper ~75 m), transitioning to Tempest below that. This approach was developed as part of the earlier

published work farther north discussed in Minsley et al 2021 (Commun Earth & Env), where interleaving Tempest and Resolve survey lines were similar in proportion. Results of that work indicate strong correspondence between the gridded/merged AEM data and existing hydrostratigraphic interpretations built from large borehole datasets (i.e. Figure 3,6, Figure S1 in that 2021 paper).

In the area of interest for this study however, the vast majority of survey lines (~90%) were flown with the Tempest system (white lines shown in map below), with only about 10% Resolve along a few river paths (red lines in map below). Given the small portion of Resolve lines and their location relative to the primary features of interest, the impact of blending systems does not have a visible impact here—results are predominantly from the Tempest survey.

We clarified the amounts of each type of data in this study area, and added text to the end of the methods section, "This approach for blending resistivity models for the two systems was developed farther north in the lower Mississippi River valley where interleaving Tempest and Resolve flights were common[16]. However, in the study area presented here about 90% of the nearly 18,000 line-kilometers were flown with Tempest. The Resolve system was only flown along some of the rivers in southwest Louisiana, and along part of the Mississippi river upstream of New Orleans and therefore do not significantly impact the merged resistivity grids presented here."

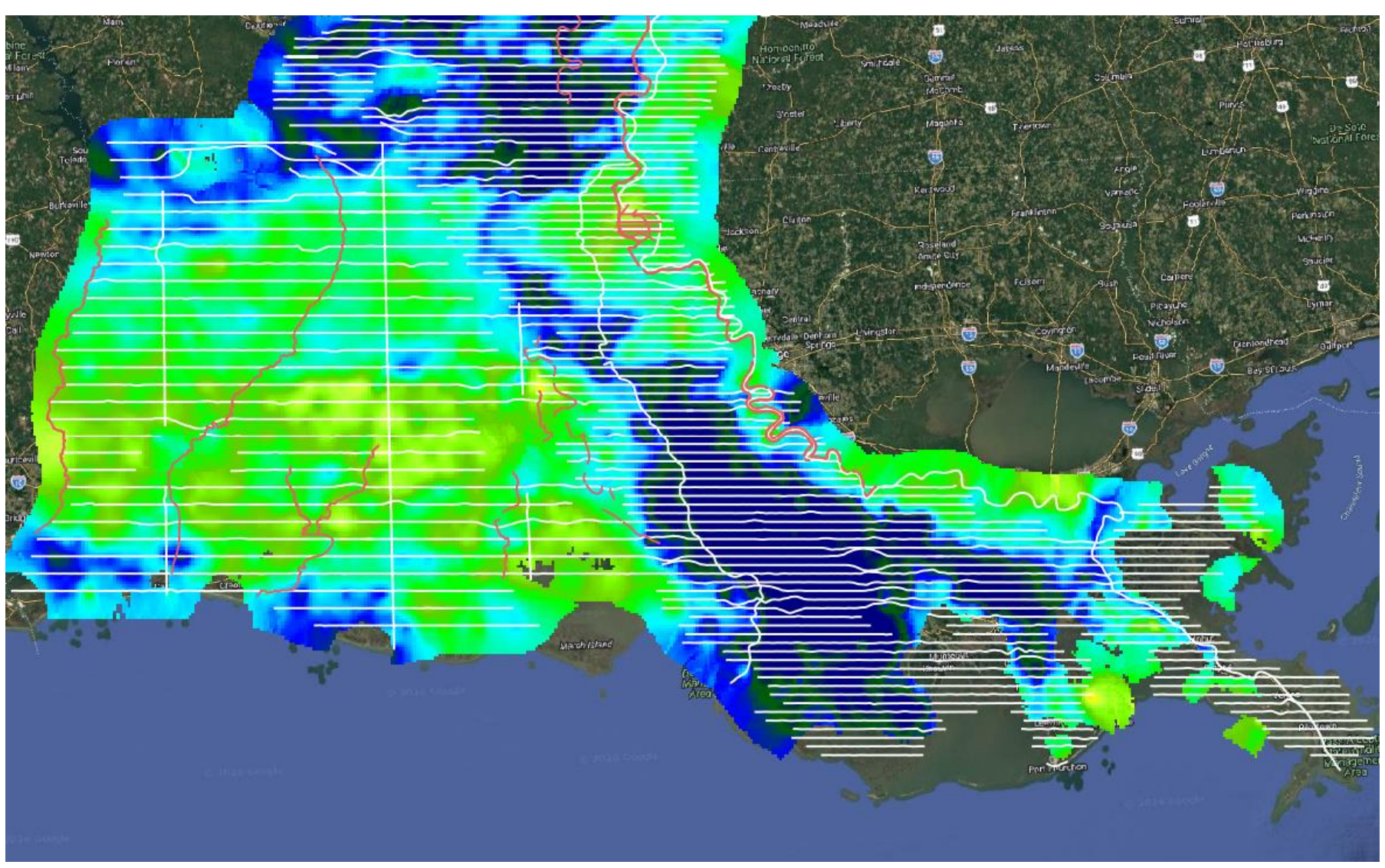

• The authors note that the buried salinity is "out of equilibrium" with modern hydrologic conditions and that flushing would be expected over time, but no estimate of flushing timescales is provided. Even an order-of-magnitude estimate or citation to relevant modeling work would help the reader assess how persistent this legacy salinity is and over what timeframe it might impact water resource management.

Added paragraph to this point that discusses results in the context of model-based studies that suggest deep saline water can persist over very long timescales in scenarios such as ours:

“Model-based studies have shown that saline water can be trapped and preserved beneath prograding delta systems over large distances and timescales in excess of 16,000 years for scenarios consistent with coastal Louisiana with rapid progradation during the Holocene, together with heterogeneous confining layers at depth[43]. Near-surface saline water introduced during Holocene transgressions can rapidly infiltrate downwards through coarse-grained incised valley fill such as the late-Pleistocene substratum, slowing where confining layers or the less permeable base of valleys is encountered[44]. Freshening of the upper substratum and Holocene topstratum occurs during progradation, leaving the saltwater legacy at depth beneath a shallow freshwater system[43,44]. Present-day hydrologic conditions would tend to flush the saline water out of the system, but the rate of basin-scale flushing decreases exponentially with depth[45], particularly in deltaic systems with characteristic heterogeneity that creates high vertical anisotropy in permeability[46,47]. Thus, the combined effects of low-permeability topstratum acting as a barrier to recharge of freshwater, deeper low-permeability layers, and the slow rate of deep groundwater flow in low hydraulic gradient coastal regions allows the saltwater to persist over glacial timescales[44–48]. “

• The paper demonstrates a compelling framework linking AEM imaging with space-for-time comparison to interpret paleo-salinity. A brief discussion of whether this approach could be applied predictively to other major delta systems globally (e.g., Mekong, Nile, Irrawaddy) where similar post-glacial histories may have left analogous buried salinity features would strengthen the paper's broader impact, particularly for the Nature Water readership.

Yes, there would likely be relevance in system-scale mapping of other coastal delta systems given the global occurrence of late-Quaternary incised valleys and Holocene initiation of marine deltas that leave an unexpected/geologically controlled saline groundwater legacy. We accordingly modified & added to the last paragraph of the main text:

“Thus, understanding the geologic history of coastal aquifers and its impact on the modern-day distribution of groundwater salinity, which may be out of equilibrium with current hydrologic conditions, is an important component of effective water resource management in populated coastal regions globally[11,12,15]. Our approach of regional-scale subsurface mapping to identify modern groundwater salinity patterns that reflect past geological processes is likely relevant to other major delta systems globally given the prevalence of late-Quaternary incised valleys[50] and the worldwide formation of marine deltas during Holocene sea level rise[51].”

• In the Methods, the DOI estimation for the Tempest data follows Oldenburg and Li (1999), while the Aarhus Workbench inversions presumably use a different DOI metric. Are these comparable? Some comment on the consistency of DOI estimates across the different systems and inversion approaches would be helpful.

Added text to the methods section describing DOI in workbench being based on the method of Christiansen and Auken (A Global Measure for Depth of Investigation., Geophysics, 2012). Both methods, as mentioned in the text, use mathematical approaches to quantify the depth to which models are constrained by observations. While different approaches implemented in different software routines, they produce comparable results. More importantly here, however, is that the depth of investigation is primarily related to the physical characteristics of different systems used (Tempest, Resolve, SkyTEM), not the DOI calculation approach. We also clarify in the Methods section about producing a combined resistivity grid from Tempest and Resolve data that the transition to 100% Tempest occurs at 75m, which is typical for the DOI of the Resolve system.

• Minor text issues: "~10,00 km^2" appears to be a typo for "~10,000 km^2" in the Delaware Bay comparison section.

fixed

Reviewer #2 (Remarks to the Author):

The authors have collected very impressive AEM datasets from Mississippi Delta and Delaware Bay. Their data clearly show a 3D image of high and low resistivity strata corresponding to saline and fresh groundwater in the Mississippi Delta (MD). They also provide a similar 3D image of resistivity in Delaware Bay to support their interpretation. They produce a very convincing model for the formation of deep saline water in the PE and CE along the Mississippi incised valley, followed by fresh water displacing the shallower saline water. I have not seen such extensive displacement of saline water documented like this. This paper is noteworthy and of broad interest, and should clearly be published. However, we have some suggests for improving the manuscript and increasing its readability that is needed before publication. The readability could be improved, perhaps because the presentation of the data and its interpretation was mingled, I needed multiple read throughs to fully understand the paper, although it was worth the effort,

Thank you for the positive feedback and suggestions, which have led to several significant modifications in the manuscript and figures. Detailed responses below

An addition that would really help for understanding the interpretation of the inversion in the MD would be a contour map of the actual incised valley, e.g., depth to base of Holocene. It would show where the incised valley is and how deeply it cut through the preexisting strata. You should be able to construct it from the Saucier and Wessels references along with other publications such as Heinrich et al., 2015 and Jankowski et al., 2017. The depth of the Holocene/Pleistocene boundary can also be added to the sections in Figure 2 e, f, g.

Following your suggestion we have included the digital information from the Heinrich et al. 2015 Holocene-Pleistocene surface. We have added the Holocene-Pleistocene surface to the Fig. 2 (and Supplementary Fig. 4) sections as recommended, as well as a new contour map in Supplementary Fig. 2. Inclusion of this surface is an important addition, as it made us reconsider the age of the shallow low-resistivity 'halo' which we previously attributed to the Holocene paleo-estuary. Based on its depth beneath the H-P surface, the revised manuscript describes this feature as a Pleistocene consolidated clay layer, supported also by borehole observations.

Note however that the Heinrich H-P surface *does not* represent the base of the late-Pleistocene incised valley but rather is recognized as the transition from coarse substratum to finer-grained topstratum at the H-P boundary. While this broadly represents the lateral extent of the valley fill, it does *not* reflect the substratum fill within the incised valley or the deepest incised channels formed in the late Pleistocene, such as shown in the figure below from Aslan and Autin, 1991. We are not aware of a source of data that contains the elevation of the base of the

substratum. We have tried to clarify this also within the text, that coarse-grained late-Pleistocene substratum fills the base of the incised valleys.

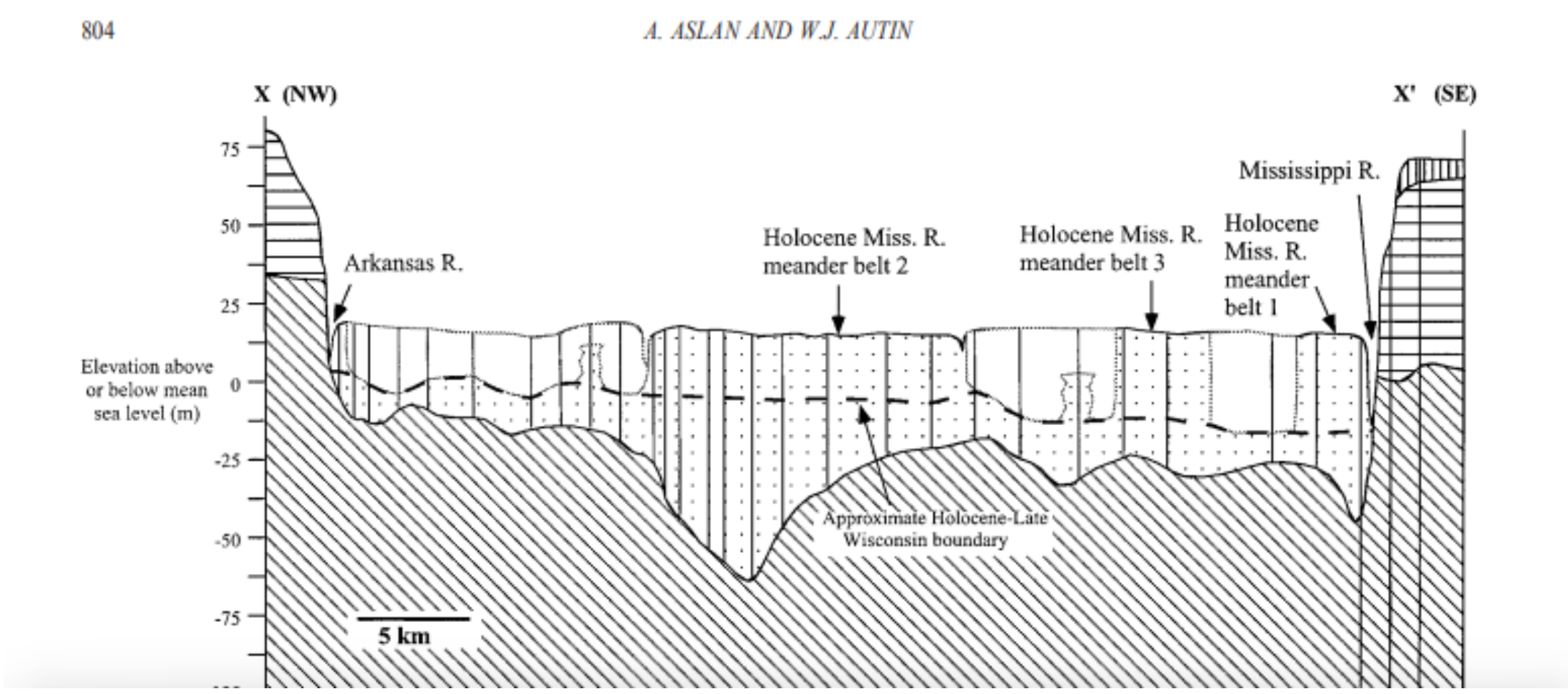

In Figure 2 add scale to the right side of the transects as well to make it easier to see the depths of the features. The map being in degrees and the section in km, also makes it harder to correlate them. I suggest adding some longitude ticks to the X axis of the sections.

Added scale to right side of all transects in Fig 2, 3, S3, S4, along with latitude ticks. Also added references where appropriate to the right side of transects linking to the elevation of corresponding map-view panels.

I agree that groundwater salinity is the primary control on conductivity, but the Extended Data Fig.1 shows a significant clay effect. A large portion of points stay within the shaded area. Therefore, I suggest specifying the resistivity range for fresh, brackish, and saline water to clarify clay effects on groundwater conductivity, perhaps as a table.

It is true that many clay-affected points inside the shaded region; however, most of these occur at relatively fresh SC/typically less than about 1000 us/cm, and found farther north in panels a-b where we also expect lower SC. This supports a similar point also raised by reviewer #1, that the clay vs salinity ambiguity dominates in fresh groundwater zones, where the presence of clay can result in multiple orders of magnitude difference in bulk resistivity as shown in the figure you reference. At moderate SC from 1000-10000, the effect of clay is less than an order of magnitude, and becomes negligible at very high SC > 10000.

We have moved the extended data figures to a separate supplementary information, with additional clarifying text. We also include there Supplementary Table 1 along the lines you suggest that communicates the increasingly limited impact of clay content as salinity increases.

On the right side of section g, there is a deeply incised saline wedge extending from the surface to over 100 m that is not discussed. I got confused as I initially thought of this as the incised

valley. It is highlighted in Figure 5. Is it a saline wedge from the abandonment of the St. Bernard lobe? Please include this in the presentation and discuss its origin.

This feature that you refer to is located beneath/at the edge of the St. Bernard lobe, and is likely a saline wedge extending in the subsurface here. However, I'm not sure if we can attribute this to the abandonment of the St. Bernard lobe, or if it is simply the steeply dipping saline wedge near the modern coast, possibly filling an earlier incised valley in that area. There are several areas where we see saline water dipping into the subsurface adjacent to coastlines, and have added text at the end of this section to highlight these,

"Saline wedges are seen dipping from the near surface near coastlines, for example in the southwest corner of Louisiana (left side of Fig. 2g), southeast of New Orleans near the edge of the St. Bernard delta lobe (right side of Fig. 2g), and along the southern coastline at the end of the valley-parallel profile Fig. 2h)."

An important part of the model is the substantial flow of PFW above the PE and CE. The areas appear to be sandy (Ex. Data Fig. 3). At the same time, you say "The present-day hydrologic conditions would tend to flush the saline water out of the system, but the presence of low-permeability materials and the slow rate of groundwater flow within deep aquifer systems allow the saltwater to persist". You have shallower parts flushed in your interpretation and deeper not. The recent Voller et al. (2025) modeling paper shows that an aquitard may be needed to produce this type of flow boundary. Can you include rough calculations about the permeability of sandy aquifers and muddy aquitards that show where the flow of fresh water could or could not displace 10s of km of saline water during the Holocene? This flow is an important part of the model shown in Figure 4. Is there any evidence of flushing such as boron isotopes in wells (Ravenscroft et al., 2004)?

This paragraph has been modified and added to, referencing the Voller et al. paper you mention, as well as Delsman et al (2014) that is relevant here and for which we add a citation and reference to. Delsman et al show that freshwater can replace saline water within decades within a permeable aquifer (such as the coarse-grained substratum here), leaving a shallow freshwater zone above the trapped deeper saline water that persists. The contact between the coarse-grained/permeable substratum and more consolidated deeper Pleistocene units could provide this contrast in hydraulic conductivity described by Voller et al—they show this behavior over a wide range of ratios in hydraulic properties from 1:20 to 1:1000, and that pattern likely exists for even smaller ratios which they do not show. Modeling studies of basin-scale flow in that same Ganges Delta system along with groundwater age dating suggest a basin-scale vertical anisotropy in permeability of 10,000:1 (Michael et al., 2009a; Michael et al., 2009b). We also point to the concept of Tothian basin-scale flow (Toth, 2016), in which nested scales of flow create vertical distributions of ages /flushing rates that increase/decay rapidly with depth.

The modified text reads, "Model-based studies have shown that saline water can be trapped and preserved beneath prograding delta systems over large distances and timescales in excess of 16,000 years for scenarios consistent with coastal Louisiana with rapid progradation during the Holocene, together with heterogeneous confining layers at depth[43]. Near-surface saline water introduced during Holocene transgressions can rapidly infiltrate downwards through coarse-grained incised valley fill such as the late-Pleistocene substratum, slowing where confining layers or the less permeable base of valleys is encountered[44]. Freshening of the upper substratum and Holocene topstratum occurs during progradation, leaving the saltwater legacy at depth beneath a shallow freshwater system[43,44]. Present-day hydrologic conditions would tend to flush the saline water out of the system, but the rate of basin-scale flushing decreases exponentially with depth[45], particularly in deltaic systems with characteristic heterogeneity that creates high vertical anisotropy in permeability[46,47]. Thus, the combined effects of low-permeability topstratum acting as a barrier to recharge of freshwater, deeper low-permeability layers, and the slow rate of deep groundwater flow in low hydraulic gradient coastal regions allows the saltwater to persist over glacial timescales[44–48]. "

Additional comments: [text is brackets are suggested additions]

Line 44 - freshwater-saltwater interface[, in the absence of other factors,] is determined by the modern equilibrium … Also add a reference.

Incorporated this suggested text, and reference to Werner et al., 2013.

"Saline and brackish groundwater occurs naturally in coastal regions, typically beneath a lens of freshwater that thickens inland. *In the absence of other controlling factors,* the position of the freshwater-saltwater interface is determined by the modern equilibrium between freshwater on land and sea level[7]"

Line 50 - led to the formation of deeply incised river valleys – provide a little more description than just "led".

Modified this sentence: "Geological processes have also altered the distribution of saline water in coastal regions globally, most notably since the last glacial maximum (LGM) approximately 20 ka when global sea level was more than 120 m below present that led to the formation of deeply incised river valleys *as river systems attempt to reach equilibrium with a new lowered base level*[8]"

Line 52 - incised valleys are filled with fluvially sediments as well as glacially derived ones.

Added "and fluvially" after "glacially"

Line 53 - These infilled valleys can be [fine-grained barriers or] permeable pathways

Changed “can be permeable pathways” to “can host permeable pathways”. Valley fill is not likely to be entirely permeable or fine grained, so wanted to communicate that permeable pathways are typically present, but not exclusive, in valley fill.

Line 119 - Why is 10 ohm-m chosen as the lower bound for the aquifer? Can you associate it with a particular salinity?

This approximate resistivity transition was chosen empirically based on observations of the range of resistivity values within the Chicot aquifer, often contrasting with deeper and much lower resistivity confining layers. Given the co-varying effects of both lithology and salinity, it is difficult to assign specific salinity values to a particular resistivity unless the lithology is known or a local regression can be made (Supplementary Fig. 1, Supplementary Table 1). Here we instead focus on the geometry of different units or high-salinity regions based on their spatial patterns and comparison with known contacts.

Line 131-2. “… which we interpret as an older paleo-estuary (PE)”. This was confusing as the PE was formed in the same transgression as is made clear a few sentences later. Perhaps: which we interpret [as a] paleo-estuary (PE) [formed by flooding of the incised valley].

Suggestion accepted.

Line 133 – How steeply dipping in the Pleistocene? They may be more steeply dipping than the coastal plain, but are probably still a pretty low dip. Either correct or provide dips.

You’re right they are all fairly low dips, though look steeper given the large vertical exaggeration on our cross-sections. The older units dip at around 4 –13 m/km, whereas the Heinrich H-P surface is ~0. 2 m/km. Modified to read:

“This low-resistivity feature continues to narrow inland until terminating in a narrow channel emerging from *more* steeply dipping low-resistivity Pleistocene and older units *(~4 – 13 m/km, compared with just ~0.2 m/km for the Holocene-Pleistocene surface)* that mark…”

Line 134 – ‘fall line’. I don’t know the usage on the Gulf Coast. Along the East Coast the fall line is at the edge of the Mesozoic coastal plain. West of the Mississippi, the appear to be two topographic breaks, one at the edge of the Mesozoic and one closer to the shore that is identified as the fall line. I don’t know which one is properly called the fall line.

Removed this phrase to avoid confusion, just referencing this as the transition to coastal plain, changed label in Fig 2d and reworded to:

“…that mark *a* transition from upland alluvial to coastal plain regions (UCT, Fig. 2d).”

Line 157 – for the moderately low-resistivity halo, perhaps a different set of initials to distinguish it from the deeper PE (e.g., PEh = PE halo or PPE = perched PE).

Based in part on your earlier comments about showing the elevation of the Heinrich Holocene-Pleistocene surface, we revised the interpretation of this shallow low-resistivity halo to be a late-Pleistocene clay layer, as it is likely too deep to be Holocene. Accordingly it is re-labeled PCL on Fig. 2e, and the text is modified here to describe this feature.

"*Supported by borehole observations at the bottom of several wells in this location*[34] *and its position beneath the H-P surface*[32]*, we* hypothesize that this low-resistivity halo represents *late-Pleistocene clays and other more consolidated sediments*. The shallow low resistivity halo and deeper salt wedge is flanked by moderate resistivity (PFW in Fig. 2e) and that we conjecture represents paleo-fresh groundwater (PFW) surrounding the saline wedge that is now preserved in the subsurface. This shallow low-resistivity halo is only evident above *the northernmost* parts of the region marked by PE in Fig. 2d; south of this location it may either have never existed, or was eroded by major floods and significant channel-filling with *coarse-grained substratum sediments*[26,29].

Line 205 – Again, the dips are not steep, although perhaps steep for GW surfaces. All the sections for MD and DB should have the same Vertical Exaggeration, which should be labeled. Fig. 3d is clearly different from e and f. The two locations are of sufficiently different scale that they cannot be easily shown with the same V.E., which is why each figure should be labeled.

Changed 'dipping steeply' to 'dipping more steeply' to make it more clear that steep is a relative term here. Modified all sections to have constant VE (200x for MD, and 25x for DB), with the VE labeled above each section.

Line 208 – In cross section view (Fig. 3c, d-f -> 3c is not a cross section.

Changed to Fig. 3d-f

Line 236 – perhaps a river valley parallel section could be added to Figure 2 or 5 to correspond to the one in Figure 4.

Added a longitudinal transect to Figure 2h.

Line 248 – Which resistivity value used here to define the top of low-resistivity feature?

It is similar to the shallow saline wedge, with values < 3-4 ohm-m. Noted that here, and also earlier in the text where this deep low-resistivity funnel is first described.

Line 248 – Why do you describe it as "multiple stages of valley filling"? I see one progressive valley filling and a flow of fresh water displacing the shallower saline water.

Changed "multiple stages of valley filling" to "distinct regions of elevated salinity related to the saline wedge left beneath previously inundated valleys" to clarify that it is not so much multiple

stages, but discrete valley fills such as the main central valley versus the area farther east that you highlight in the area of the St Bernard lobe.

Line 258 – "of poor water quality" probably better described as non-potable saline water.

Accepted this change

Line 278 - Another recent imaging of a paleovalley by EM is Le et al., 2025.

We included this reference here and discussed earlier in the text in response to comments from reviewer #1.

Figure 2 - The unit of specific conductance can be converted to resistivity (ohm.m) for direct comparison to resistivity values from AEM, even though they measure different properties (bulk resistivity versus pore fluid). Add IV and TS in f and PE in g. Plot all sections (e, f, g) at the same scale

True the specific conductance could be communicated as ohm-m; however, since these data are widely reported in us/cm we chose to keep these units in order to maintain familiarity with what people are used to.

Added IV to panel f. With the addition of the Holocene-pleistocene surface on all sections, it should be clear that the TS is generally above this curve. Added CE to panel g.

All sections plotted with 200x vertical exaggeration for coastal Louisiana (here and in Supplementary Fig. 4)

Figure 3. Typo in 'Open Water Extent" in legend. Again use the same scales in the 3 sections. The confining layers likely bend upwards under the bay, as the sediment loading would depress them on the flanks. It is likely that some confining layers correspond to the boundary between the PFW inflow and the high resistivity below.

Fixed typo in legend. All sections plotted with 25x vertical exaggeration for Delaware Bay (and in Supplementary Fig. 3). We have left these conceptual confining layer lines as they are, without having evidence for them bending upwards beneath the bay and left as dashed to show they are inferred.

Figure 4. Typo in a: perpendicular. Present-day coastline in a should align with section g and be at top of section. Add PWF for areas where fresh water has displaced salt water. Are the dashed dark blue lines schematic mixing flow in salt water wedge? This should be indicated in the legend.

Fixed typo in perpendicular. Moved present-day coastline annotation as recommended. Added PFW and other annotations in g,h. Yes, the dark blue lines are meant to indicate circulation in

the salt water wedge, and follows the light-dark blue fresh-saline colorscale. Also modified this figure by removing the overlay blue color scheme for saline water throughout the cross-sections, which masked the geology-based colors. Instead, the region of high salinity is outlined in red and with fine stipple, with colored arrows indicating fresh/saline water flow.

Figures 1, 2, 3, 5 label some of the water depth contours.

Added depth contour labels to these, and the supplementary figures.

Extended Data Figure 1. Why is a significant amount of clay-dominated lithology not within the shaded area, where surface conduction of clay is required to explain conductivity of sediments not obeying Archie's law? Using the same unit: ohm.m or S/m would be better for this demonstration. Or could have two x-axis labels. Which is Archie's law equation or version that you use to infer this? Need references and specifying parameters used in the equation, such as temperature. You might consider adding a table to classify specific groups: freshwater- vs saltwater-regions, clay outside the shaded region versus sand inside the shaded region. I found this graph is useful to interpret freshwater/saltwater distribution and clay effects. However, you should mention its limitations such as not considering temperature and the mismatch between the resolution scale between AEM and SC.

A primary reason that there are many clay-dominated points outside the shaded region is that while surface conduction is *necessary* to explain points in the shaded region, the presence of clay does not guarantee that a point *must* fall in the shaded region. For a given fluid salinity, the interval between the shaded region and the straight line representing no surface conduction represents valid bulk resistivity values where bulk resistivity is decreased by some surface conduction—but not so much that it falls below the resistivity of the pore fluid itself. These clay points outside the shaded region may just indicate some amount of clay, but not sufficient surface conduction to drop the bulk resistivity below the fluid resistivity. As you suggest scale differences between the AEM gridded resistivity and SC measurements could also affect this result where clay-dominated measurements are mixed with higher resistivity lithology. We have added this as additional text to the Supplementary Information supporting Supplementary Fig. 1. We believe the incorporation of surface conduction effects due to the presence of clays is an important factor in this work, and is not utilized in other studies such as the work by Le et al. cited in this review who only use Archie's law without considering surface conduction.

While correct that fluid resistivity varies with temperature, the decrease in resistivity is only about 2% for each one degree Celsius increase [Sen, P. N., & Goode, P. A. (1992). Influence of temperature on electrical conductivity on shaly sands. *Geophysics*, 57(1)]. So, if the resistivity at 20C was 50 ohm-m, the corresponding resistivity at 10C would be 60 ohm-m – a subtle difference (about 0.08 in log-resistivity units) compared with the regional-scale variability we

observe in resistivity. We note in the supplementary text that temperature is not expected to be a major controlling factor on resistivity here.

Also added a second x-label on top of the plot.

Figure 5. You could add the GW age values above sections b, c to help compare the map and section view.

A good suggestion- though I believe you are referring to Supplementary Figure 3—we have added the age values on sections b & c in this figure.

References

Heinrich, P., Paulsell, R., Milner, R., Snead, J. & Peele, H. Investigation and GIS development of the buried Holocene-Pleistocene surface in the Louisiana coastal plain. p.140 Louisiana Geological Survey and Louisiana State University for the Louisiana Coastal Protection and Restoration Authority (2015).

Jankowski, K. L. et al. (2017) Vulnerability of Louisiana's coastal wetlands to present-day rates of relative sea-level rise. Nat. Commun. 8, 14792 doi: 10.1038/ncomms14792.

Le, H.D., K. Key, M.S. Steckler, N. Sazeed, M. Person, A. Bhuiyan, M.R. Khan and K.M. Ahmed (2025). Buried deep freshwater reserves beneath salinity-stressed coastal Bangladesh, Nature Comm., 16, 10740, https://doi.org/10.1038/s41467-025-65770-4.

Ravenscroft P. and J.M McArthur, 2004, Mechanism of regional enrichment of groundwater by boron: the examples of Bangladesh and Michigan, USA, Applied Geochemistry, 19, 1413-1430, https://doi.org/10.1016/j.apgeochem.2003.10.014.

Voller, V. R., Sazeed, N., Luong, L., Camille, A., Steckler, M., Leary, K., & Person, M. (2025). Onshore entrapment of seawater in coastal aquifers by rapid coastline progradation. Water Resources Research, 61, e2024WR038157. https://doi.org/10.1029/2024WR038157

Reviewer #3 (Remarks to the Author):

I co-reviewed this manuscript with one of the reviewers who provided the listed reports. This is part of the Nature Communications initiative to facilitate training in peer review and to provide appropriate recognition for Early Career Researchers who co-review manuscripts.

Thanks for your co-review!

Reviewer #4 (Remarks to the Author):

Dear editor and authors,
The paper describes paleo-saline groundwater volumes for two (large) study areas (large buried coastal paleo-estuaries) along the coast of the USA, based on large-scale airborne electromagnetic measurements (AEM). I've read the paper with pleasure but the findings are very site specific and the findings for general scientific interest are not novel.
The main findings as summarized in the abstract are (literally cited):

We are glad you liked the paper, but sorry to hear you do not think the findings are overly site specific and insufficiently novel. We address your specific comments below.

Finding 1: "Coastal groundwater management typically focuses on preventing intrusion from modern sources of seawater; however, past geological processes can also leave a legacy of saline groundwater now hidden in the subsurface.".
This is common sense and well known for many delta's in the world like the Dutch delta and the Nile delta. Aquifers of these delta's contain paleo-saline groundwater infiltrated during their geological development and transgressions in the Holocene and earlier inter-glacial periods. Nowadays, they face major challenges in groundwater management for freshwater availability for agriculture and drinking water.

While the concept of past geology controlling modern salinity is no doubt known, a major component of the challenges faced in groundwater management is that a system-scale snapshot of these aquifer systems is unavailable due to observational limitations. In some of the prominent examples cited here, for example Le et al. (2025) or Larsen et al (2017), the role of geophysics in identifying modern salinity controlled by past geology in coastal Bangladesh and the Red River, respectively, is demonstrated. Also site-specific, these studies base their conclusions in part on spatially limited geophysical datasets collected with sites along a transect within large delta regions. While useful in providing subsurface information, these types of ground-based studies cannot produce system-scale information needed to characterize vast regions—an important advance that we demonstrate in this manuscript. As just you suggest, many of these regions face major challenges in groundwater management; however, it is difficult to manage what is not measured.

- Finding 2: "Here we show, for the first time using multiple extensive airborne electromagnetic (AEM) surveys, detailed evidence of the residual salinity from a paleo-estuary filling a late-Pleistocene incised valley impacting more than 10,000 km2 that is now hidden beneath coastal Louisiana's deltaic plain."
This is very site specific, though a nice example, besides many others. Airborne electromagnetic

(AEM) surveys for large scale mapping the salinity of the subsurface have been carried out since the beginning of this century and is a well known and proven method published in many papers.

We dispute that this is a very site-specific study given that it is one of the largest AEM studies published globally for groundwater salinity investigations, covering over half the state of Louisiana and including a second example in this synthesis from Delaware Bay. All of the previously published AEM studies for groundwater salinity in the US would only cover a small fraction of the area presented here. Our manuscript does not claim to be the first to use AEM in salinity investigations, but rather the first to demonstrate its use for system-scale mapping of basin-scale salinity effects, and to draw novel conclusions about the deep salinity impacts from past marine transgressions based on a modern drowned river valley in a different geological setting. Studies about specific places are widespread in the literature; here, we document novel findings about groundwater salinity—an important factor in coastal groundwater management—in one of North America's largest incised valley systems by building on the established developments in the use of AEM.

- Finding 3: "Our results show that the three-dimensional pattern of saline groundwater beneath Louisiana mimics that of near-surface aquifers surrounding the modern Delaware Bay estuary, fingerprinting the signature of the past drowning of a large, incised valley of the Mississippi River following post-glacial sea-level rise.
This finding is very site specific, see comments for finding 2.

We are unaware of any restrictions in Nature Communications from demonstrating results about specific places, as opposed to global syntheses or other generic theoretical or modeling developments. Rather, it is necessary to describe the results from specific places where unique observations have been made (AEM in this case) to develop new insights into the hydrogeologic history that impacts modern water resources. We further highlight the global relevance of our findings by adding to the end the manuscript, "Our approach of regional-scale subsurface mapping to identify modern groundwater salinity patterns that reflect past geological processes is likely relevant to other major delta systems globally given the prevalence of late-Quaternary incised valleys[46] and the worldwide formation of marine deltas during Holocene sea level rise[47]."

Numerous publications in Nature Communications, not to mention other similar high-impact journals, have focused on site-specific studies that relate to the paleo-history of fresh and saline water and groundwater management. These articles document both methodological advances and process understanding through demonstration at focused study areas, and are a valuable complement to other non-site-specific model-based or synthesis studies.

Le, H., Key, K., Steckler, M.S. et al. Buried deep freshwater reserves beneath salinity-stressed coastal Bangladesh. Nat Commun 16, 10740 (2025). https://doi.org/10.1038/s41467-025-65770-4

Micallef, A., Person, M., Haroon, A. *et al.* 3D characterisation and quantification of an offshore freshened groundwater system in the Canterbury Bight. *Nat Commun* **11**, 1372 (2020). https://doi.org/10.1038/s41467-020-14770-7

Sheng, C., Jiao, J.J., Luo, X. *et al.* Offshore freshened groundwater in the Pearl River estuary and shelf as a significant water resource. *Nat Commun* **14**, 3781 (2023).

Liu, PW., Famiglietti, J.S., Purdy, A.J. *et al.* Groundwater depletion in California's Central Valley accelerates during megadrought. *Nat Commun* **13**, 7825 (2022). https://doi.org/10.1038/s41467-022-35582-x

- Finding 4: "These findings demonstrate a new framework for understanding legacy sources of saltwater critical for managing stressed water resources along global coastlines."
There is no new framework demonstrated. They demonstrated for 2 study areas the use of AEM in combination with ground-truth data to map and understand the paleo-saline groundwater occurrences.

We did not present two distinct case studies but rather sought to synthesize understanding of subsurface salinity beneath Delaware Bay, a modern drowned river valley, to bring insight into the saline legacy beneath a paleo drowned-river valley in coastal Louisiana. We are not aware of this sort of multi-survey synthesis of AEM studies to conduct this sort of space-for-time understanding. Likewise, there have been no AEM studies published in North America (and only a few globally) at the scale of our study in coastal Louisiana. By opening the aperture of our measurements to the entire hydrogeologic system covering tens of thousands of square kilometers, we are able to gain insight beyond what is available from limited site-scale studies.

To summarize, the findings presented in the paper are site specific and the general ideas and applied methodology cannot be judged as novel. It is common sense that paleo-saline groundwater occurrences below deltas resulted from past transgressions and occur widespread globally being critical for freshwater availability. Using large scale AEM for mapping those paleo saline groundwater bodies is regularly done since the past 15 years. Therefore, I will not recommend publication in Nature.

Review responses below in blue
Burke Minsley
13 July 2026

REVIEWER COMMENTS

REVIEWERS' COMMENTS

Reviewer #2 (Remarks to the Author):

The authors edited to text to respond to our comments, and those of the other reviewers. The manuscript addressed our issues and is now improved and easier to read. I just had a few additional minor suggested changes and now recommend the article for publication.

Thank you for this follow-up review.

Change:
This low-resistivity feature continues to narrow inland until terminating in a narrow channel emerging from more steeply dipping low-resistivity Pleistocene and older units (~4–13 m/km, compared with just ~0.2 m/km for the H-P surface) that mark an interpreted 'fall line' (FL) marking the transition from upland alluvial to coastal plain regions (d).
To:
This low-resistivity feature continues to narrow inland until terminating at the interpreted 'fall line' (FL) which marks the upland alluvial to coastal plain transition (UCT; dashed line). This coincides with a change from low-resistivity Pleistocene units farther north dipping at (~4–13 m/km, compared with just ~0.2 m/km for the H-P surface.
Since the FL and UCT are on the map, but the dips are not,identify with those first, then add the dip comments.

We've made this change in order of these phrases, however reference to the 'fall-line' had already been removed due to confusion with that term in the original revision (you may have seen this in the tracked-changes?). Also removed reference to FL in the figure 2 caption and replaced with UCT. This now reads: "*This low-resistivity feature continues to narrow inland until terminating in a channel emerging from Pleistocene and older units that mark a transition from upland alluvial to coastal plain regions (UCT, dashed line, Fig. 2b-d). This transition coincides with a change from the low-resistivity Pleistocene units farther north that have steeper dip (~4–13 m/km) compared with the H-P surface (~0.2 m/km).*"

Change:
This inferred paleo-estuary may have formed during the early stages of marine transgression that first filled deeply entrenched channels in this region33 before continued sea-level rise filled the coastal embayment (CE) that occupies a much larger area of nearly 10,000 km2.
To:
This inferred paleo-estuary may have filled during the early stages of marine transgression33 before continued sedimentation during sea-level rise filled the coastal embayment (CE) that occupies a much larger area of nearly 10,000 km2.
It is the sediments, not the SLR that fill the estuary

Change accepted

Better to call TS topstratum and then describe it as a clay cap, that say clay cap (TS).

Changed to: "Moderately low resistivity in the near-surface correlates with the *topstratum* (TS) *that forms a clay cap* over much of the deltaic plain"

Figure 2. Label of profile h is missing. Explanation of PCL and UCT are missing from caption.

Added label for profile h, and explanations of PCL and UCT to the caption.

Reviewer #3 (Remarks to the Author):

I co-reviewed this manuscript with one of the reviewers who provided the listed reports. This is part of the Nature Communications initiative to facilitate training in peer review and to provide appropriate recognition for Early Career Researchers who co-review manuscripts.

Thank you for this follow-up review.

Reviewer #4 (Remarks to the Author):

Based on the authors' response, the positive assessments provided by the other reviewers, and the overall quality of the manuscript, I no longer consider my earlier concern regarding the site-specific nature of the study to constitute a valid argument against publication.
The extensive spatial scale of the AEM survey, which facilitates the derivation of system-scale insights—particularly with respect to the development of one of North America's largest incised

valley systems—renders the study both scientifically robust and broadly relevant. I agree with the authors' response that it is necessary to describe results from specific places in order to make unique observations, which are essential for developing new insights.
In light of these considerations, I support the publication of this manuscript in Nature Communications.

Thank you for this follow-up review and re-consideration.
